# Proteome expansion in the *Potyviridae* evolutionary radiation

**DOI:** 10.1093/femsre/fuac011

**Published:** 2022-02-23

**Authors:** Fabio Pasin, José-Antonio Daròs, Ioannis E Tzanetakis

**Affiliations:** Instituto de Biología Molecular y Celular de Plantas (IBMCP), Consejo Superior de Investigaciones Científicas-Universitat Politècnica de València (CSIC-UPV), 46011 Valencia, Spain; School of Science, University of Padova, 35121 Padova, Italy; Instituto de Biología Molecular y Celular de Plantas (IBMCP), Consejo Superior de Investigaciones Científicas-Universitat Politècnica de València (CSIC-UPV), 46011 Valencia, Spain; Department of Entomology and Plant Pathology, Division of Agriculture, University of Arkansas System, 72701 Fayetteville, AR, USA

**Keywords:** *Potyviridae*, virus comparative genomics, non-core proteome module, evolutionary radiation, host adaptation, immune evasion

## Abstract

*Potyviridae*, the largest family of known RNA viruses (realm *Riboviria*), belongs to the picorna-like supergroup and has important agricultural and ecological impacts. Potyvirid genomes are translated into polyproteins, which are in turn hydrolyzed to release mature products. Recent sequencing efforts revealed an unprecedented number of potyvirids with a rich variability in gene content and genomic layouts. Here, we review the heterogeneity of non-core modules that expand the structural and functional diversity of the potyvirid proteomes. We provide a family-wide classification of P1 proteinases into the functional Types A and B, and discuss pretty interesting sweet potato potyviral ORF (PISPO), putative zinc fingers, and alkylation B (AlkB)—non-core modules found within P1 cistrons. The atypical inosine triphosphate pyrophosphatase (ITPase/HAM1), as well as the pseudo tobacco mosaic virus-like coat protein (TMV-like CP) are discussed alongside homologs of unrelated virus taxa. Family-wide abundance of the multitasking helper component proteinase (HC-pro) is revised. Functional connections between non-core modules are highlighted to support host niche adaptation and immune evasion as main drivers of the *Potyviridae* evolutionary radiation. Potential biotechnological and synthetic biology applications of potyvirid leader proteinases and non-core modules are finally explored.

## Teaser

*Potyviridae* represents one of the most notable evolutionary radiations among RNA viruses; its species richness is accompanied by large genomic layout diversity and expansion of the encoded protein modules.

## Introduction

Understanding the origin and evolution of viruses is complex, yet it is fundamental to fully realize the ecological, agricultural and medical impact of the virosphere (Jones and Naidu 2019, Zimmerman *et al*. 2020, Holmes *et al*. 2021, Liang and Bushman 2021). Plant virus diseases are major threats to food security; they occur worldwide and greatly affect developing countries (Jones and Naidu 2019, Savary *et al*. 2019). Conceptual frameworks rationalize the polyphyletic origins and evolution of the plant virome, as well as its ecological impact on crops and wild species (Lefeuvre *et al*. 2019, Dolja, Krupovic and Koonin 2020). Genomic resources for plant viruses have increased in the past four decades (Pasin, Menzel and Daròs 2019), but our knowledge of plant virus evolution and host adaptation mechanisms is nonetheless incomplete.

**Figure 1.**
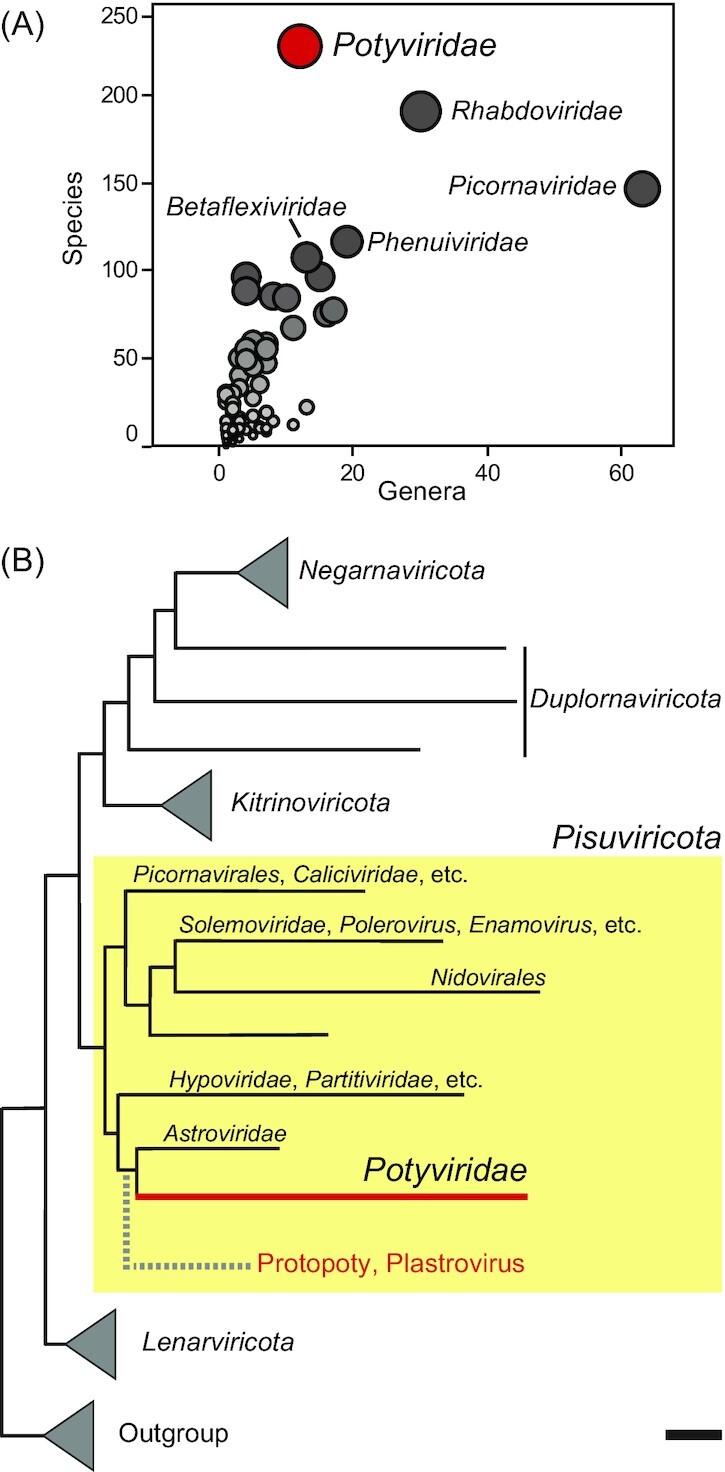
*Potyviridae* within the known RNA virosphere. **(A)** Species and genus abundance in the recognized families of the realm *Riboviria*; families with > 100 species are labeled (see Table S1 of Supporting Information). **(B)** Schematic phylogenetic tree of the RNA virus RNA-dependent RNA polymerases (RdRp). RNA virus phyla are indicated, and the branches of *Pisuviricota* are expanded; reverse transcriptases of group II introns and non-long-terminal-repeat transposons were used as an outgroup; scale bar = 0.5. The overall tree topology was taken from Wolf *et al*. (2018), and updated to include the virus lineages (red text) with reported ancestral status to *Potyviridae* (Lauber *et al*. 2019, Wolf *et al*. 2020).

The plant-infecting *Potyviridae* is the largest RNA virus family (realm *Riboviria*) (Fig. 1A). The most recent virus taxonomy based on phylogenomic analyses places the family within the phylum *Pisuriviricota* (Fig. 1B), which comprises of members of the former picorna-like supergroup (Koonin *et al*. 2020). Potyvirid genomes are a mosaic of modules with polyphyletic origins that can be linked to multiple unrelated viruses, either within and outside *Pisuriviricota* (Dolja, Krupovic and Koonin 2020, Gibbs *et al*. 2020). Despite their complex origin, emergence and diversification of modern potyvirids have been traced to plant-associated astro-like viruses (plastroviruses) and protopotyviruses, groups of viruses identified in plant transcriptomes and aquatic samples (Lauber *et al*. 2019, Wolf *et al*. 2020) (Fig. 1B).

*Potyviridae* includes > 200 plant virus species currently assigned to the twelve genera *Arepavirus*, *Bevemovirus*, *Brambyvirus*, *Bymovirus*, *Celavirus*, *Ipomovirus*, *Macluravirus*, *Poacevirus*, *Roymovirus*, *Rymovirus*, *Tritimovirus*, and *Potyvirus*, with this last being the most speciose (Gibbs *et al*. 2020, International Committee on Taxonomy of Viruses 2020). Potyvirids have positive single-stranded RNA genomes of 8-11 kb that are translated into polyproteins, which are in turn hydrolyzed by viral proteinases to release a set of mature products (Adams, Antoniw and Beaudoin 2005, Revers and García 2015). First studies of potyvirid genomes identified a basic layout with conserved gene abundance and order. Yet recent discoveries spurred by sequencing technological advances have revealed a large variability in the genomic structures and gene content. Potyvirid polyproteins indeed show a common core led by diversified leaders that are enriched in non-core modules which expand the proteome structural and functional heterogeneity.

Here, we present a pan-family survey of the structural and functional diversity of the *Potyviridae* proteomes by delineating core and non-core modules (see Supporting Information). We provide a family-wide classification of P1 proteinases, and review knowledge of non-core domains. We examine abundance of the leader helper component proteinase (HC-pro) within the family, and uncover a putative papain-like protease domain in polyprotein leaders of known and putative *Celavirus* members. Using non-core module evolution as a case study, we summarize main molecular mechanisms that have acted in the *Potyviridae* radiation.

We also discuss the finding that common immune evasion roles can be identified in potyvirid leader cistrons and those of plant, fungal and animal viruses; pointing to host adaptation as a main driver of their evolution. A perspective on the applications of potyvirid leader proteinases and other non-core modules in biotechnology and synthetic biology is also presented.

## Core and non-core modules of *Potyviridae* proteomes

**Figure 2.**
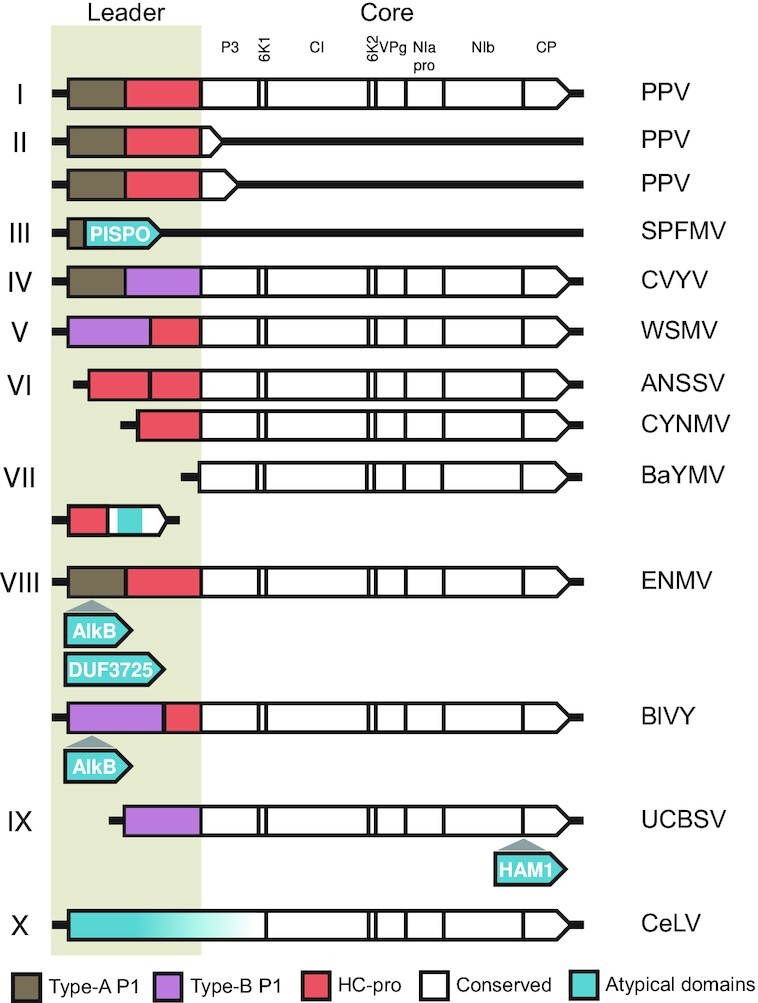
Layout and leader diversity of *Potyviridae* polyproteins. RNA molecules and encoded polyproteins are represented as lines and arrowed boxes, respectively; the hypervariable *N* termini (leaders), and the conserved middle and *C*-terminal (core) regions are indicated. Representative layouts are as follows: I, Type-A P1 and HC-pro in full-length polyproteins; II, Type-A P1 and HC-pro in truncated polyproteins generated by P3 frameshifting (P3N-PIPO, and P3N-ALT); III, PISPO production by frameshifting in Type-A P1 cistrons; IV, tandem of Type-A and Type-B P1; V, Type-B P1 and HC-pro; VI, single or tandem copies of HC-pro, and P1 absence; VII, leader-less RNA1, and additional RNA2; VIII, Type-A or Type-B P1 including alkylation B (AlkB) or DUF3725; IX, inosine triphosphate pyrophosphatase (HAM1) in polyproteins with Type-B P1 and no HC-pro; X, atypical leader with non-conserved domains. Diagrams are for illustrative purposes and not to scale; PPV, plum pox virus; SPFMV, sweet potato feathery mottle virus; CVYV, cucumber vein yellowing virus; WSMV, wheat streak mosaic virus; ANSSV, areca palm necrotic spindle–spot virus; CYNMV, Chinese yam necrotic mosaic virus; BaYMV, barley yellow mosaic virus; ENMV, endive necrotic mosaic virus; BlVY, blackberry virus Y; UCBSV, Ugandan cassava brown streak virus; CeLV, celery latent virus.

Genera of *Potyviridae* have a common polyprotein core which is expanded by a heterogeneous array of non-core modules (Fig. 2). A set of eight mature proteins is conserved in the middle and carboxy (*C*) terminus of the polyproteins, namely P3, 6 kDa protein 1 (6K1), cytoplasmic inclusion (CI) protein, 6 kDa protein 2 (6K2), viral genome-linked protein (VPg), nuclear inclusion protein A proteinase (NIa-pro), nuclear inclusion protein B (NIb), and coat protein (CP) (Revers and García 2015). P3N-PIPO and P3N-ALT are generated by a frameshifting mechanism in the P3 cistron, and are conserved (Yang, Li and Wang 2021, Choi *et al*. 2022). These conserved proteins have a common but polyphyletic origin (Gibbs *et al*. 2020). NIa-pro and NIb are homologous to picorna-like signature genes, being, respectively, a chymotrypsin-like cysteine proteinase and an RNA-dependent RNA polymerase (RdRp) with phylogenetic affinity to animal-infecting *Astroviridae* and other *Pisuriviricota* members. CP was likely acquired from other filamentous RNA viruses, whereas CI is a superfamily 2 helicase most closely related to flavivirid homologs (Koonin *et al*. 2008, Zamora *et al*. 2017, Dolja, Krupovic and Koonin 2020).

Organization of polyprotein amino (*N*) termini (leaders) is highly variable and bears distinctive genus- or even species-specific features (Fig. 2). Protein hidden Markov model (HMM) profiles allow for sensitive homology detection and have been applied to infer evolution of viral proteomes, as well as virus identification in metatranscriptomic datasets and taxonomic assignment (Nasir and Caetano-Anollés 2015, Wolf *et al*. 2018, Bin Jang *et al*. 2019, Callanan *et al*. 2020). A combination of HMM and protein profile scans was applied here to quantitatively survey the diversity and abundance of the *Potyviridae* non-core modules. P1 and HC-pro are the most common, but not universal, leader cistrons (Yang, Li and Wang 2021). Other non-core modules identified in few potyvirid species include the pretty interesting sweet potato potyviral ORF (PISPO), putative zinc fingers and DUF3725, alkylation B (AlkB), inosine triphosphate pyrophosphatase (ITPase/HAM1), as well as a pseudo tobacco mosaic virus-like coat protein (TMV-like CP) domain.

## Diversity and evolution of non-core modules

### P1 proteinases—two phylogenetically and biochemically distinct lineages

**Figure 3.**
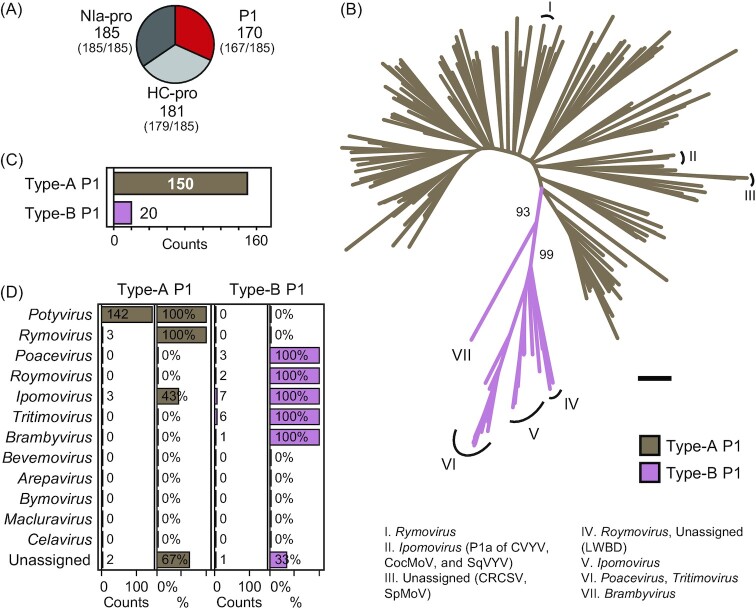
Family-wide phylogeny and abundance of Type-A and Type-B P1 lineages. **(A)** Abundance of the potyvirid-encoded proteinases P1, HC-pro and NIa-pro. Ratio of species with at least one domain of the indicated proteinase *vs*. total species is shown in parentheses (Table S2). **(B)** P1 phylogeny. Protease domain sequences were aligned (Figure S1), and phylogeny was inferred (Supporting Methods); numbers beside branches indicate bootstrap support values; scale bar = 1. All *Potyvirus* branches are unlabeled and are included in the Type-A lineage; branches of remaining genera are labeled. The ipomoviruses cucumber vein yellowing virus (CVYV), *Coccinia* mottle virus (CocMoV), squash vein yellowing virus (SqVYV) encode a Type-B homolog and an additional Type-A copy (branch II). Common reed chlorotic stripe virus (CRCSV), *Spartina* mottle virus (SpMoV), and longan witches' broom-associated virus (LWBD) are orphans. **(C)** Family counts of Types A and B. **(D)** Abundance of Types A and B across genera of *Potyviridae*. Absolute numbers (Counts) and counts per species (%) are shown. Unassigned includes CRCSV, SpMoV, and LWBD.

P1 is the least abundant among the potyvirid proteinases (Figs 2 and 3A). The *C* terminus includes a well-conserved chymotrypsin-like serine protease domain, a common module of RNA viruses, which autocatalytically releases P1 from the polyprotein (Rodamilans *et al*. 2018, Mann and Sanfaçon 2019). The *N* terminus is hypervariable, intrinsically disordered and dispensable for P1 proteolysis (Valli, López-Moya and García 2007, Pasin, Simón-Mateo and García 2014). It can tolerate sequence insertions and diverse atypical domains and functional motifs can be found within it (Fig. 2). *Potyvirus* P1 is active *in planta* and in plant-based translation systems but its proteolysis is very low or absent in animal systems (Rohožková and Navrátil 2011). This supports the hypothesis that activation of potyviral P1 requires a plant co-factor, the identity of which is yet unknown.

Family-wide phylogenesis of the conserved protease domain supports the presence of two distinct lineages—Types A and B (Fig. 3B). Type A is predominant (88%; Fig. 3C), and includes homologs that display plant co-factor dependency in *in vitro* cleavage assays; it is found in all members of *Potyvirus* and *Rymovirus*, and in 3/7 of ipomoviruses (Fig. 3D). Type-B proteinases do not need plant co-factors, displaying robust self-processing in multiple translation systems including bacteria (Rodamilans, Valli and García 2013, Shan *et al*. 2018). This lineage is found in all *Ipomovirus*, *Roymovirus*, *Poacevirus*, *Tritimovirus*, and *Brambyvirus* members (Fig. 3D). A tandem of both lineages is found in the ipomoviruses cucumber vein yellowing virus (CVYV), squash vein yellowing virus (SqVYV), and *Coccinia* mottle virus (CocMoV) (Figs 2, 3B and D) (Dombrovsky, Reingold and Antignus 2014, Desbiez *et al*. 2016).

Type-A P1 acts as a viral accessory factor, since deletion mutants are infectious and capable of replication and systemic movement (Rohožková and Navrátil 2011, Pasin, Simón-Mateo and García 2014). Consistent with its dispensability, ∼10% of the recognized potyvirid species lack P1 (see *Arepavirus*, *Bevemovirus*, *Bymovirus*, *Celavirus*, and *Macluravirus* of Fig. 3D).

### Pretty interesting sweet potato potyviral ORF (PISPO)

The potyvirus sweet potato feathery mottle virus (SPFMV) has a large Type-A P1 and defective HC-pro (Yang, Li and Wang 2021). Transcriptional slippage takes place within P1 with the derived transcripts coding for a truncated P1 and the frameshift protein PISPO, which participates in RNA silencing suppression (Fig. 2, and see below) (Mingot *et al*. 2016, Untiveros *et al*. 2016). Besides SPFMV, PISPO is present in sweet potato virus 2, C, and G (Clark *et al*. 2012).

### Zinc fingers and DUF3725

Zinc-finger domains mediate interaction with DNA, RNA, and proteins, and have a variety of cellular functions that include antiviral immunity regulation. A divalent cation coordinates two cysteines and histidines in CCHH zinc-fingers, but different cysteine/histidine compositions are found in the non-canonical CCHC, CCCH, and CCCC zinc-fingers, all of which have reported RNA interacting ability (Cassandri *et al*. 2017, Corley, Burns and Yeo 2020, Wang and Zheng 2021).

**Figure 4.**
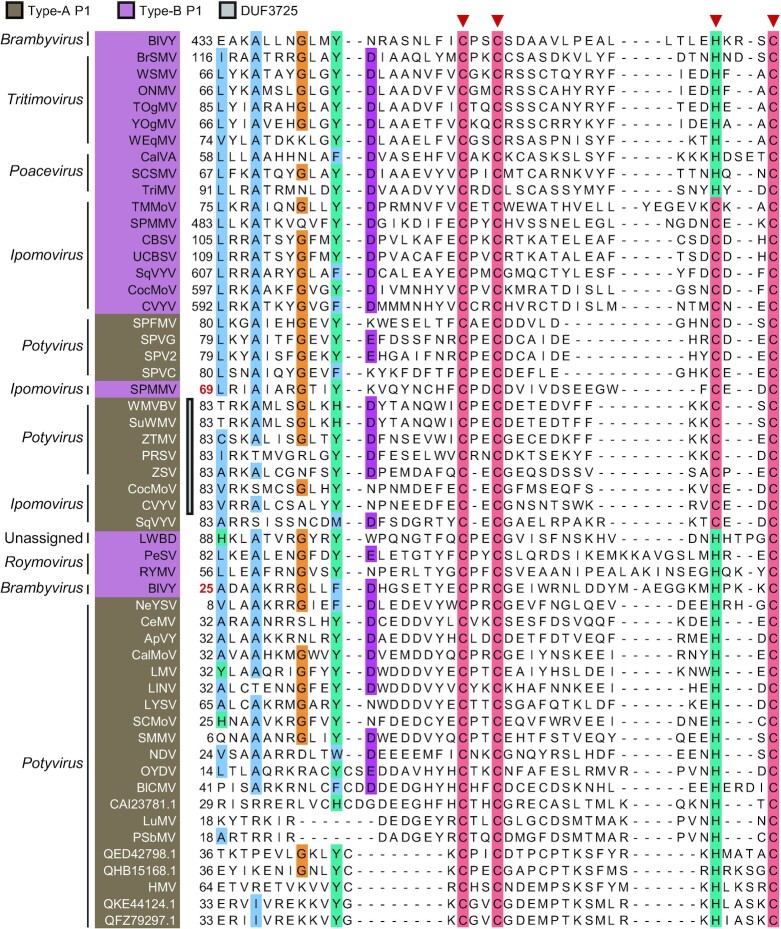
Putative zinc-finger motifs and DUF3725 in P1. Alignment of a conserved cysteine-rich region of Type-A and Type-B P1s is shown; inverted triangles indicate putative zinc-finger residues described to be involved in RNA silencing suppression activity of Type-B homologs (Valli, Dujovny and García 2008, Kenesi *et al*. 2017, Gupta and Tatineni 2019a). For each virus, position of the first aligned polyprotein residue is indicated, and colored in red to label duplications; accession numbers are shown or given in Table S2 alongside virus complete names.

Putative CCCC or CCHC zinc fingers are present in all known Type-B P1s, and are found with varying degrees of conservation in the *N* terminus of many Type-A orthologs (Fig. 4). Duplicated P1 zinc fingers can be identified in blackberry virus Y (BlVY; *Brambyvirus*) and sweet potato mild mottle virus (SPMMV; *Ipomovirus*). Conserved CCCC and CCHC motifs of Type-B P1s were functionally linked to RNA silencing suppression (Valli, Dujovny and García 2008, Kenesi *et al*. 2017, Gupta and Tatineni 2019a). Consistently, zinc fingers are known in diverse RNA silencing suppressors of plant viruses (Csorba, Kontra and Burgyán 2015, Sõmera, Sarmiento and Truve 2015).

Type-A P1s lack silencing suppression activity and biological roles of their putative zinc fingers remain unknown. The CCCC motif in some of them partially overlaps DUF3725 (Pfam: PF12523) (Fig. 4). DUF3725 is found in *Streptomyces* bacteriophage proteins (ATE85218.1) that share similarities to the zinc-binding domain of DnaG-like primases, which coordinates template binding and RNA primer synthesis in the replication of double-stranded DNA viruses (Gao *et al*. 2019).

### Alkylation B (AlkB)

AlkB domains are ubiquitous among prokaryotes and eukaryotes; having iron(II)- and *α*-ketoglutarate-dependent dioxygenase activity that reverses nucleic acid methylation damage (Fedeles *et al*. 2015).

**Figure 5.**
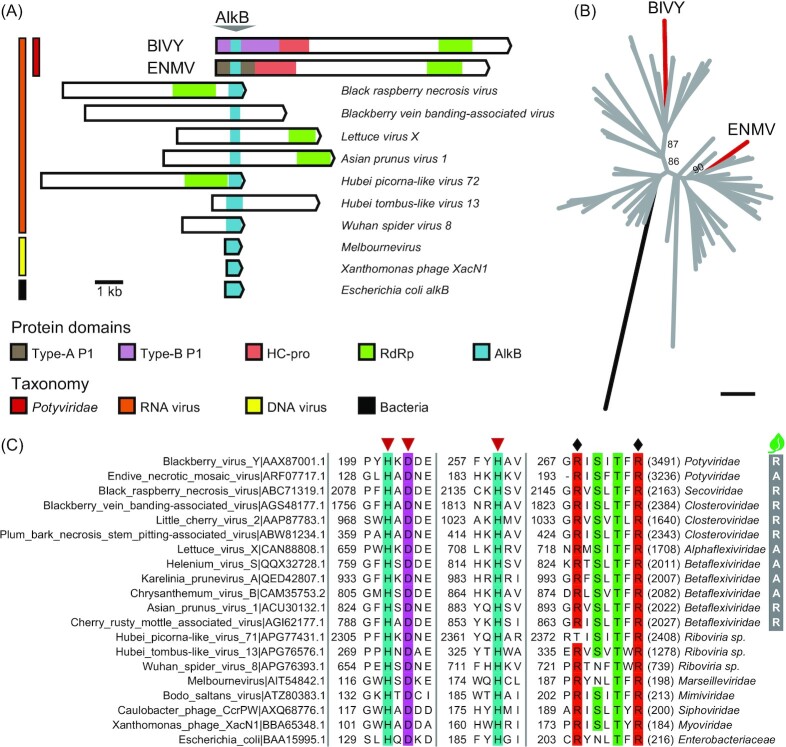
AlkB in *Potyviridae* and divergent virus taxa. **(A)** Diagrams of representative ORF bearing AlkB; relevant domains are colored. Species and taxonomic groups (left) are labeled; BlVY, blackberry virus Y (genus *Brambyvirus*); ENMV, endive necrotic mosaic virus (genus *Potyvirus*); *Escherichia coli alkB* as a standard. **(B)** AlkB phylogeny. Protein sequences in plant viruses were aligned (Figure S2), and phylogeny was inferred; numbers beside branches indicate bootstrap support values; scale bar = 1; *E. coli* AlkB was included as a reference (black); *Potyviridae* accessions are labeled (red). **(C)** Conserved residues in viral AlkB proteins. Alignment blocks show regions of *E. coli* AlkB that participate in catalysis (inverted triangles) or *α*-ketoglutarate binding (diamonds) (Yu *et al*. 2006). Position of the first residue is indicated (left), and the (poly)protein size is shown in parentheses. Right, virus taxonomic groups and plant host families are indicated (R, Rosaceae; A, Asteraceae).

AlkB is found in atypical P1s of endive necrotic mosaic virus (ENMV; *Potyvirus*) and BlVY (*Brambyvirus*; Fig. 5). AlkB of the latter catalyzes the *in vitro* removal of RNA methyl groups (van den Born *et al*. 2008). Hypermethylation of viral RNA genomes negatively affects plant and animal cell infection (Martínez-Pérez *et al*. 2017, Zhang, Qian and Jia 2021). AlkB was suggested to safeguard viral genomic integrity through repair of methylation damage and promote long-term infection of perennial hosts (van den Born *et al*. 2008, Martínez-Pérez *et al*. 2017). A plant AlkB domain inserted within the tobacco etch virus (TEV) genome was rapidly lost and it did not confer any fitness benefit (Willemsen *et al*. 2017), detailed characterization of AlkB roles in potyvirid infection remains to be addressed.

AlkB is embedded within replication-associated proteins of plant RNA viruses in the families *Alphaflexiviridae*, *Betaflexiviridae*, *Closteroviridae*, and *Secoviridae* (Fig. 5). Phylogenomic analysis of plant viruses has highlighted a divergent evolutionary history for AlkB compared to other viral protein domains; it was concluded that AlkB probably emerged by multiple independent acquisition events (Bratlie and Drabløs 2005). For example, the divergent genomic organization and significant phylogenetic separation of BlVY and ENMV suggests that the two viruses acquired the domain independently (Fig. 5B). BlVY and ENMV have been identified in plants of the Rosaceae and Asteraceae, respectively (Susaimuthu *et al*. 2008, Desbiez *et al*. 2017), known to host several AlkB-encoding viruses (Fig. 5C). Mixed infections are common in plants, and the AlkB origin in potyvirids can possibly be traced to independent events of inter-family gene transfer.

AlkB is distributed across divergent taxonomic groups of RNA and DNA viruses that include invertebrate RNA viruses (Shi *et al*. 2016), DNA bacteriophages (Yoshikawa *et al*. 2018), and giant DNA viruses (Fig. 5). A complex DNA methylation landscape was observed in genomes of the last of these (Jeudy *et al*. 2020), and viral AlkB may have roles in its regulation.

### Inosine triphosphate pyrophosphatase (ITPase/HAM1)

ITPase is widespread in cellular organisms, hydrolyzing triphosphates of non-canonical purine nucleotides to prevent their incorporation in nucleic acids and preserve genome integrity (Simone, Pavlov and Borgstahl 2013).

**Figure 6.**
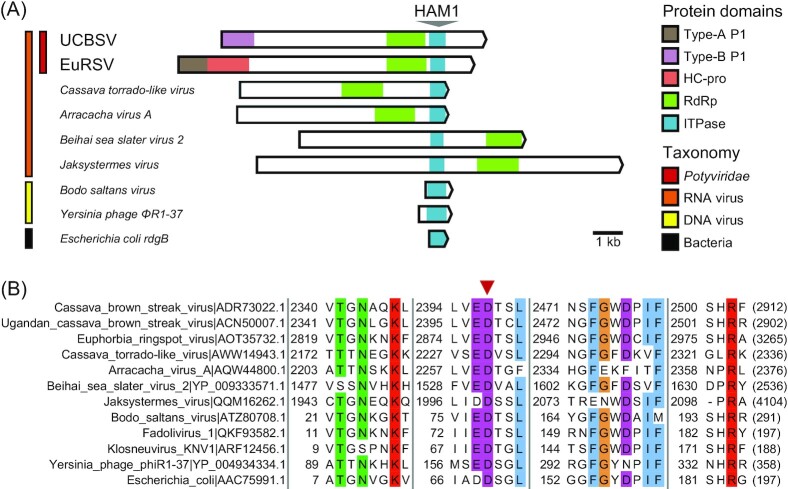
HAM1/ITPase in *Potyviridae* and divergent virus taxa. **(A)** Diagrams of representative viral ORF including the inosine triphosphate pyrophosphatase (ITPase/HAM1) fold; relevant domains are colored. Left, taxonomic groups and species are shown; UCBSV, Ugandan cassava brown streak virus (*Ipomovirus*); EuRSV, *Euphorbia* ringspot virus (*Potyvirus*); *E. coli* ITPase *rgdB* is included as a standard. **(B)** Conserved residues in ITPase sequences. Alignment blocks show regions of *E. coli* RdgB that participate in substrate binding or catalysis (inverted triangle) (Savchenko *et al*. 2007). Position of the first residue is indicated (left), and (poly)protein size is shown in parentheses.

A viral ITPase, also known as HAM1, was first identified in Ugandan cassava brown streak virus (UCBSV) and cassava brown streak virus ( CBSV) (Figs 2 and 6). The two are atypical ipomoviruses that lack canonical Type-A P1 and HC-pro, encoding a single Type-B P1 with RNA silencing suppressor activity (Mbanzibwa *et al*. 2009, Dombrovsky, Reingold and Antignus 2014, Alicai *et al*. 2016, Shan *et al*. 2018). ITPase was later identified in *Euphorbia* ringspot virus (EuRSV; *Potyvirus*), encoding Type-A P1 and HC-pro (Fig. 6A) (Knierim, Menzel and Winter 2017). CBSV ITPase, although not essential for infection of experimental hosts, was involved in viral accumulation and symptom development. Contrary to the predicted antimutagenic activity of ITPase, viral mutation rates were not reduced in transgenic plants overexpressing CBSV ITPase, nor they were increased in CBSV clones lacking ITPase (Tomlinson *et al*. 2019). Use of improved sequencing approaches and alternative experimental systems could help shed light on the ITPase roles in potyvirid infection.

Metagenomics surveys have uncovered ITPase across diverse RNA and DNA virus taxa (Fig. 6). The ITPase fold is found in plant and invertebrate RNA viruses (Shi *et al*. 2016, Le Lay *et al*. 2020, Leiva *et al*. 2022), as well as in bacteriophages and giant DNA viruses (Kiljunen *et al*. 2005, Deeg, Chow and Suttle 2018, Sun and Ku 2021).

### Tobacco mosaic virus-like coat protein (TMV-like CP)

*Bymovirus* is the only potyvirid genus whose member transmission is mediated by soil-borne plasmodiophorids (Jiang *et al*. 2020). Bymoviruses have bipartite genomes with RNA1 encoding the potyvirid polyprotein core, and RNA2, which encodes a second polyprotein processed in P2-1 and P2-2 (You and Shirako 2010). P2-1 is closely related to HC-pro (see below). P2-2 shares no similarity with other potyvirid proteins, and bymoviruses with its truncation or complete deletion are able to replicate and systemically move, but could not be transmitted by the natural vector (You and Shirako 2010).

**Figure 7.**
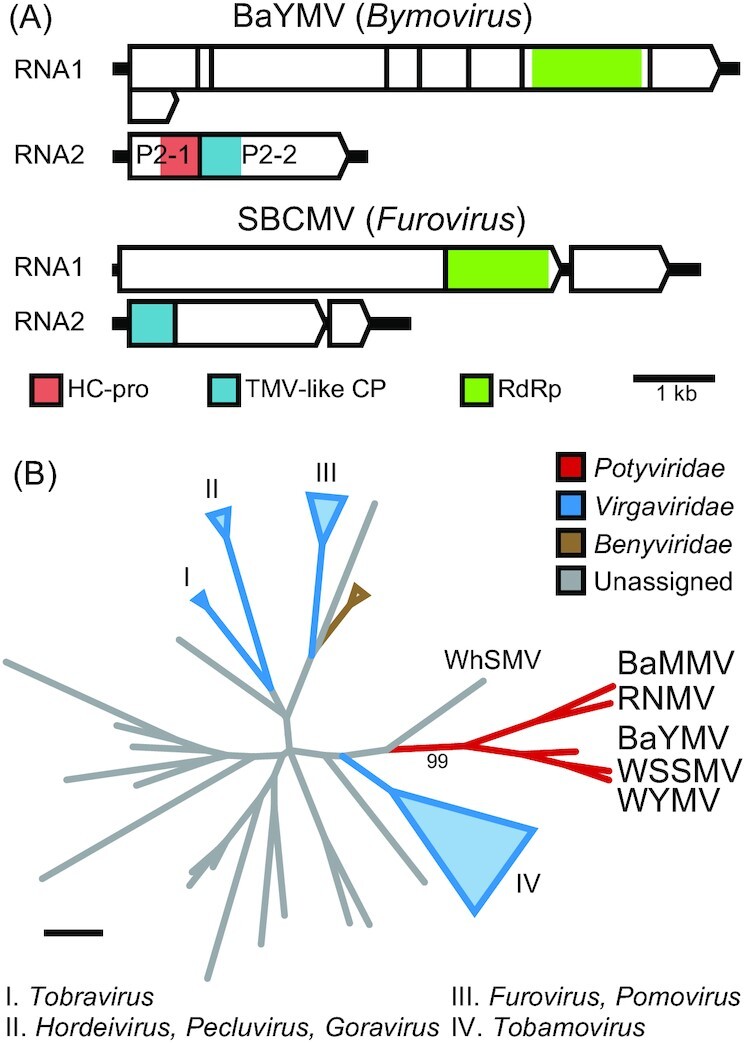
Monophyletic origin of the pseudo TMV-like CP domains of *Bymovirus*. **(A)** Genomic diagrams of the potyvirid barley yellow mosaic virus (BaYMV; genus *Bymovirus*) and soil-borne cereal mosaic virus (SBCMV; *Furovirus*), a *Virgaviridae* vectored by soil-borne plasmodiophorids. Relevant domains are colored; TMV-like CP, tobacco mosaic virus-like coat protein domain. BaYMV RNA2 encodes P2-1, with HC-pro homology, and P2-2, with a conserved ‘pseudo’ TMV-like CP domain. **(B)** TMV-like CP phylogeny of bymoviruses and reference RNA viruses. Protein sequences were aligned (Figure S3), and phylogeny was inferred; number beside branches indicates the bootstrap support value; scale bar = 1. Bymovirus accessions are in red—barley mild mosaic virus (BaMMV), rice necrosis mosaic virus (RNMV), wheat spindle streak mosaic virus (WSSMV), wheat yellow mosaic virus (WYMV). Wheat stripe mosaic virus (WhSMV) is a putative benyvirus (Valente *et al*. 2019).

Plasmodiophorid-transmitted viruses include *Virgaviridae* and *Benyviridae* members (Tamada and Kondo 2013), whose capsid proteins show homology with bymovirus P2-2 (Dessens and Meyer 1996) (Fig. 7A). HMM-profile scans detect a ‘pseudo’ TMV-like CP domain conserved in P2-2 of all full-length bymovirus accessions and absent in oat mosaic virus, whose reference sequence is of a mechanically propagated isolate which lacks most of P2-2 (You and Shirako 2010). TMV-like CP sequences of bymoviruses cluster within a monophyletic clade, which supports their common origin (Fig. 7B). The P2-2 domains show phylogenetical relatedness to CP of *Virgaviridae*, and of wheat stripe mosaic virus (WhSMV), a putative benyvirus (Fig. 7B). Besides plant viruses, the TMV-like CP fold is found in algae and invertebrate RNA viruses but has negligible homology with cellular proteins (Nasir and Caetano-Anollés 2015, Shi *et al*. 2016, Vlok, Gibbs and Suttle 2019).

### Helper component proteinase (HC-pro)

**Figure 8.**
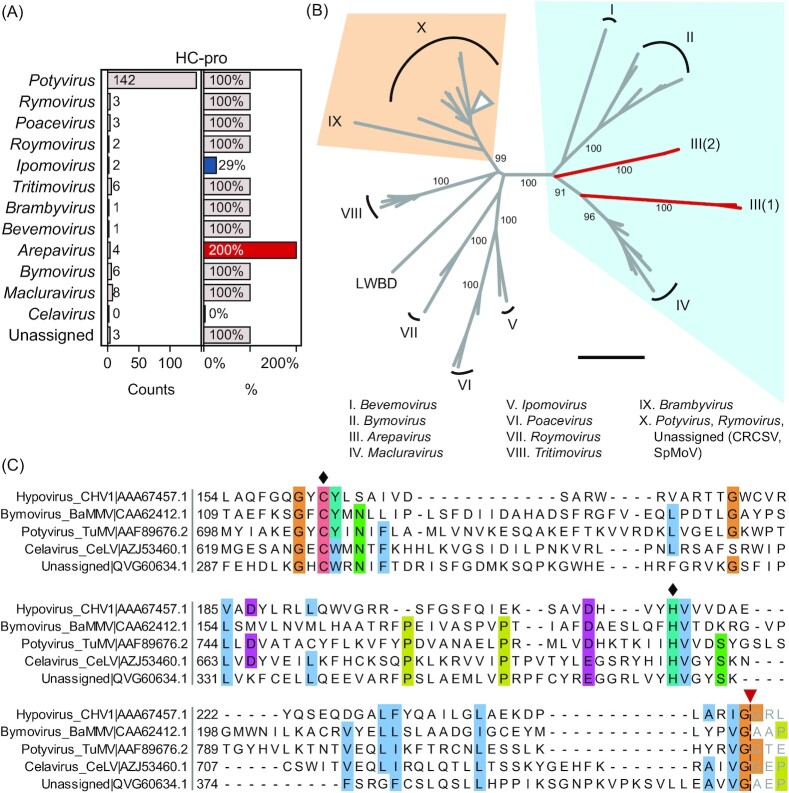
HC-pro and papain-like protease domains in *Potyviridae*. **(A)** HC-pro homologs across genera of *Potyviridae*. Absolute numbers (Counts) and counts per species (%) are shown (Table S2). Unassigned includes CRCSV, SpMoV, and LWBD. **(B)** HC-pro phylogeny. Protease domain sequences were aligned (Figure S4), and phylogeny was inferred; number beside branches indicates the bootstrap support value; scale bar = 1. Roman numerals indicate genera; branch II includes *Bymovirus* P2-1; *Arepavirus* members encode an HC-pro tandem and branches of the first and second encoded copies are labeled with III(1) and III(2), respectively; LWBD, CRCSV, SpMoV are orphans. **(C)** Putative HC-pro-like domain in *Celavirus*. Alignment of papain-like protease domains of celery latent virus (CeLV; *Celavirus*) and *Striga* potyvirus B (QVG60634.1), with turnip mosaic virus (TuMV) HC-pro, barley mild mosaic virus (BaMMV) P2-1, and *Cryphonectria* hypovirus 1 (CHV1) p29; polyprotein accession numbers and positions are indicated; diamonds, catalytic residues; inverted triangle, known or putative cleavage sites.

HC-pro is a multifunctional leader proteinase with roles in virus transmission, polyprotein processing, and suppression of antiviral RNA silencing (Valli *et al*. 2018). The HC-pro RNA silencing suppressor activity is indispensable for potyvirus infection (Garcia-Ruiz *et al*. 2015), and, based on current data, it can be safely considered a *Potyvirus* core component. Within a family-wide perspective, the reported genomic variation beyond the genus *Potyvirus* supports the HC-pro classification as a non-core module. Several ipomoviruses naturally lack HC-pro and its sequence is absent in ∼3% of potyvirid genomes (Figs 3A and 8A). Experimental evidence using a clone of wheat streak mosaic virus (WSMV; *Tritimovirus*) with complete HC-pro deletion shows the protein is dispensable for virus replication and movement (Stenger, French and Gildow 2005). Tritimoviruses as well as ipomoviruses enlist Type-B P1 as the viral silencing suppressor (Valli, Dujovny and García 2008, Mbanzibwa *et al*. 2009, Giner *et al*. 2010, Young *et al*. 2012). HC-pro is thus dispensable in potyvirids that encode proteins evolved to take over key functions originally described for homologs of model potyviruses. Adaptive HC-pro functional loss and dependency evolution were reported for onion yellow dwarf virus, which encodes a defective HC-pro *trans*-complemented by a co-infecting potyvirus (Jayasinghe *et al*. 2021). Bymovirus RNA1 lacks leader proteases, whereas RNA2 encodes the HC-Pro homolog P2-1 (Adams, Antoniw and Beaudoin 2005), two functionally and phylogenically divergent HC-pro copies are present in arepaviruses (Qin *et al*. 2020) (Fig. 8A and B).

HC-pro has a papain-like cysteine protease domain that autocatalytically hydrolyzes its *C* terminus (Guo, Lin and Ye 2011), and shows significant sequence divergence within the family that can be possibly rooted close to the bevemovirus ortholog and bymovirus P2-1 (Fig. 8B). HC-pro shows homology to the nsP2 main proteinase of alphaviruses, as well as leader proteinases of closteroviruses, picornaviruses and arteriviruses (Gorbalenya, Koonin and Lai 1991, Mann and Sanfaçon 2019). Homology identification between HC-pro and *Cryphonectria* hypovirus 1 (CHV1) p29 was instrumental to postulate the evolutionary relationship between *Potyviridae* and *Hypoviridae*, a family of fungal RNA viruses (Koonin *et al*. 1991) (Figs 1B and 8C). Papain-like cysteine proteases are common in cellular organisms, and main components of plant immunity (Misas-Villamil, van der Hoorn and Doehlemann 2016).

### Celery latent virus—an outlier

*Celavirus* is a single-member genus with celery latent virus (CeLV) as the largest and most divergent of recognized potyvirids (Gibbs *et al*. 2020). CeLV polyprotein initiates with a signal peptide that could translocate reporter proteins to the endoplasmic reticulum (Rose *et al*. 2019), no other *N*-terminal signal peptides are known in potyvirids. Sensitive HMM-profile scans failed to identify P1, HC-pro, or other potyvirid non-core modules. Inspection of the CeLV leader nonetheless reveals the presence of a putative papain-like protease domain with sequence similarity to HC-pro and bymovirus P2-1, as well as p29 of the hypovirus CHV1 (Fig. 8C). The identified catalytic residues and cleavage site are conserved in *Striga* potyvirus B (QVG60634.1; Fig. 8C), a virus phylogenetically related to CeLV and recently reported as *Striga*-associated poty-like virus 2 (Choi *et al*. 2022).

## Mechanisms of non-core module evolution

**Figure 9.**
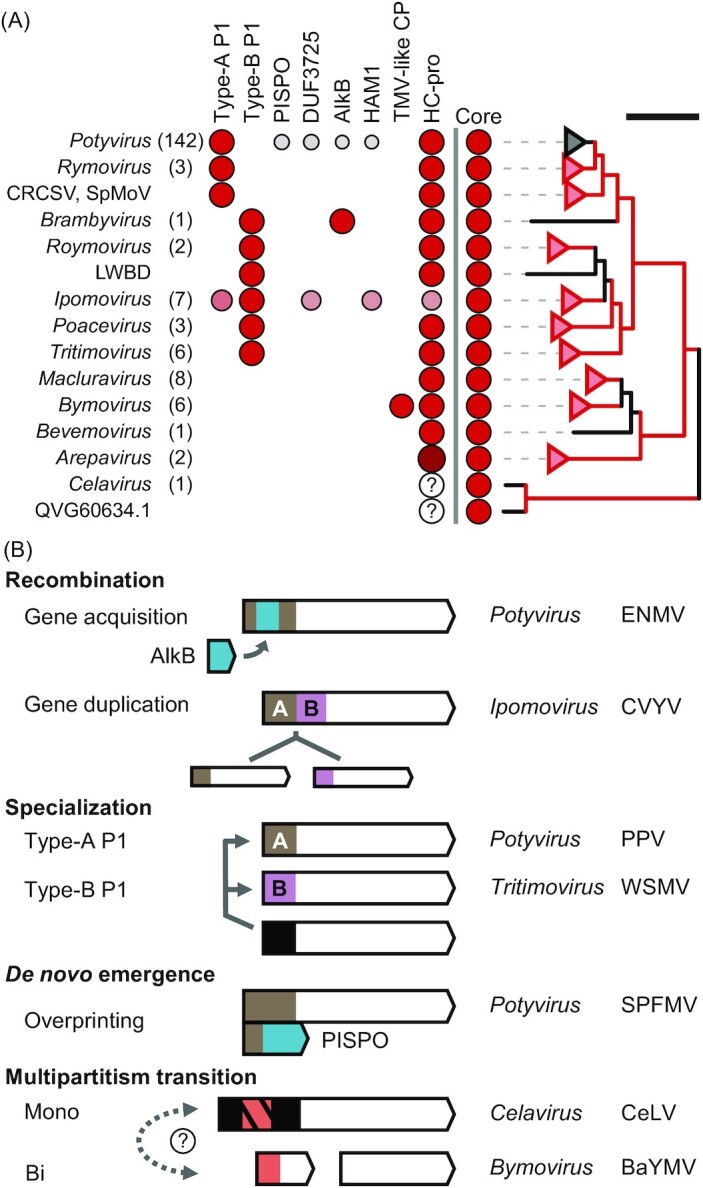
*Potyviridae* non-core proteome diversity and evolution drivers. **(A)** Abundance of non-core proteome components in genera of *Potyviridae* and recognized orphan species (CRCSV, SpMoV, LWBD); species numbers per genus are in parentheses; QVG60634.1, *Striga* potyvirus B; question marks indicate presence of putative homologs. Right, family phylogeny; RdRp domains were identified, protein sequences were aligned (Figure S5), and phylogeny was inferred; branches with bootstrap support ≥ 95 are in red, scale bar = 1. **(B)** Mechanisms and examples of non-core component evolution in *Potyviridae*. Non-core module inventory and virus complete names are given in Table S2.

Our pan-family, quantitative survey of the *Potyviridae* proteomes defines the abundance of non-core modules and highlights discrete distribution patterns along the evolutionary tree of the family (Fig. 9A). High mutation rates, recombination, gene duplication and *de novo* emergence as well as extensive gene loss and gain, and host-niche adaptation drive virus evolution. Which are the main molecular mechanisms behind non-core proteome expansion in the family's evolutionary radiation?

Recombination is common in RNA viruses and an important component of potyvirid speciation (Sztuba-Solińska *et al*. 2011). Its significance in potyvirid non-core module evolution can be clearly exemplified by AlkB identification in the potyvirus ENMV and the brambyvirus BlVY (Fig. 9B), possibly linked to independent acquisition events occurred in mixed infections with unrelated plant viruses.

Gene duplication is a major source of phenotypic novelty in cellular organism (Innan and Kondrashov 2010). It is however rare in RNA viruses, with the *Closteroviridae* coat protein duplication as a notable exception in plant viruses (Simon-Loriere and Holmes 2013). Tandem P1 or HC-pro copies in ipomoviruses and arepaviruses, respectively, were related to duplication events (Valli, López-Moya and García 2007, Qin *et al*. 2020). Empirical results nonetheless show that redundant sequences are rapidly purged from potyvirid genomes despite the potentially beneficial effect of the encoded proteins. Artificial insertion of a second HC-pro copy in the genome of TEV was deleterious and rapidly lost (Willemsen *et al*. 2016). Experimental evolution of a PPV clone encoding its own Type-A P1 and a second ortholog from a phylogenically distant potyvirus led to an array of progeny viruses with enhanced fitness that were characterized by an almost or complete duplication loss (Rodamilans, Casillas and García 2021). Together the results indicate that both sequence identity and functional redundance constrain gene duplication in potyvirids. Further supported by the polyphyletic origin of duplicated copies (e.g. see *Ipomovirus*-encoded P1s labeled by II and V in Fig. 3), it can be concluded that gene duplication events detected in potyvirids are likely by-products of interspecific, ortholog recombination (Fig. 9B).

Neofunctionalization and functional specialization in potyvirids can be inferred from biochemical and biological characterization of P1 lineages (Fig. 9B). P1 was identified as a host adaptation determinant based on gene swapping and infection assays, and on genome-wide analysis of nucleotide variation (Salvador *et al*. 2008, Maliogka *et al*. 2012, Shan *et al*. 2015, Nigam *et al*. 2019), at a protein level, it shows conserved structural disorder (Pasin, Simón-Mateo and García 2014). Structurally flexible segments in viral proteins increase mutation tolerance and adaptability through acquisition of new linear motifs or protein domains (Gitlin *et al*. 2014, Charon *et al*. 2018, Mishra *et al*. 2020). Strong evolvability and adaptation capacities of P1 are corroborated by family-wide identification of heterogenous motifs and domains within the P1 *N* termini, as well as the *de novo* emergence of PISPO through overprinting. Subfunctionalization allows the division of functions in duplicated genes (Innan and Kondrashov 2010). A zinc finger motif is conserved in all Type-B proteins but absent in most of P1s (Fig. 4); subfunctionalization of a Type-B-like ancestor could have participated in Type-A specialization.

Recently proposed scenarios place the bisegmented bymoviruses at the evolutionary diversification root of potyvirid genera, which are suggested to have originated through genomic segment fusion (Qin *et al*. 2020). Celaviruses have monopartite genomes (Rose *et al*. 2019, Choi *et al*. 2022), and their ancestral status compared to bymoviruses as supported by RdRp phylogeny makes direction of the multipartitism transition uncertain. Experimental examples are known of transitions from an originally non-segmented virus to a bisegmented one (Lucía-Sanz and Manrubia 2017). Supported by identification of a conserved pseudo TMV-like CP in bymoviruses, an intriguing possibility to explain the bipartidism emergence is the recruitment by a monopartite ancestor of a new genomic segment from co-infecting tobamoviruses to access to a vector transmission mode unprecedented within the family; transfer of the HC-pro homolog P2-1 could have been required to stabilize this *de novo* association and the gained multipartite state.

## Immune evasion through leader and non-core modules of potyvirids and other RNA viruses

In microbial systems, large taxonomic variability can be summarized by sets of redundant, polyphyletic functions (Louca *et al*. 2018). Functional analyses of cellular pangenomes suggest that gain of non-core genes influences adaptation of plant microbes to ecological niches (Box 1). Notwithstanding the low level of structural and biochemical conservation: Do *Potyviridae* non-core modules share biological function(s) and a common evolutionary driver? We present evidence supporting a main biological role of these non-core modules in counteracting host defensive reactions and thus host adaptation.

### Potyvirid leader modules and RNA silencing evasion

Plant RNA viruses have evolved a variety of strategies to modulate disease severity and escape cellular antiviral responses (Paudel and Sanfaçon 2018, Li and Wang 2019, Križnik, Baebler and Gruden 2020). Being the first translation products, viral leader cistrons are considered important virulence and pathogenicity factors that can coordinate the early infection stages.

**Figure 10.**
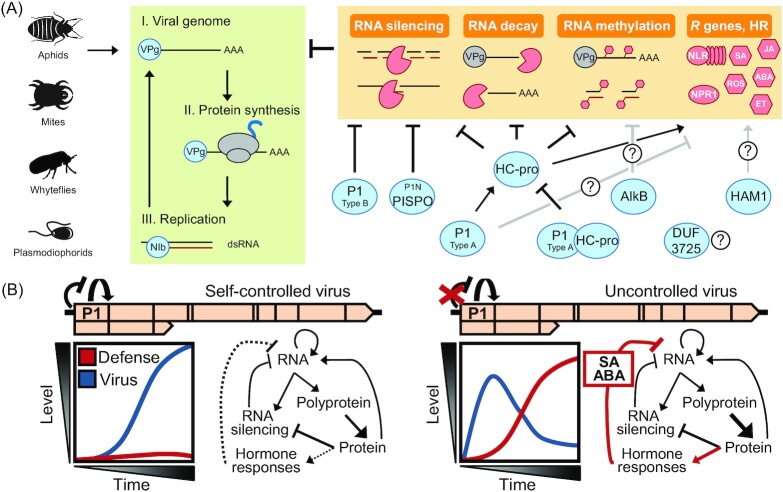
Immune evasion roles of non-core modules of *Potyviridae*. **(A)** Diagram of *Potyviridae* infection and replication stages, as well as main antiviral pathways in which non-core modules participate. Question marks indicate roles with unknown mechanisms or unavailable *in vivo* validation data; HR, hypersensitive response; NLR, NOD-like receptor; NPR1, NONEXPRESSOR OF PATHOGENESIS-RELATED GENES 1; SA, salicylic acid; ROS, reactive oxygen species; JA, jasmonic acid; ABA, abscisic acid; ET, ethylene. **(B)** Immune evasion by virus post-translational negative autoregulation. Left, P1 autoinhibition allows PPV virulence attenuation and evasion of defense responses; right, removal of the P1 autoinhibitory domain leads to an accelerated PPV amplification that activates hormone responses, which restrict virus accumulation in the long term (Pasin *et al*. 2020). Plots depict levels of virus accumulation (blue) and defense response activation (red).

**Figure 11.**
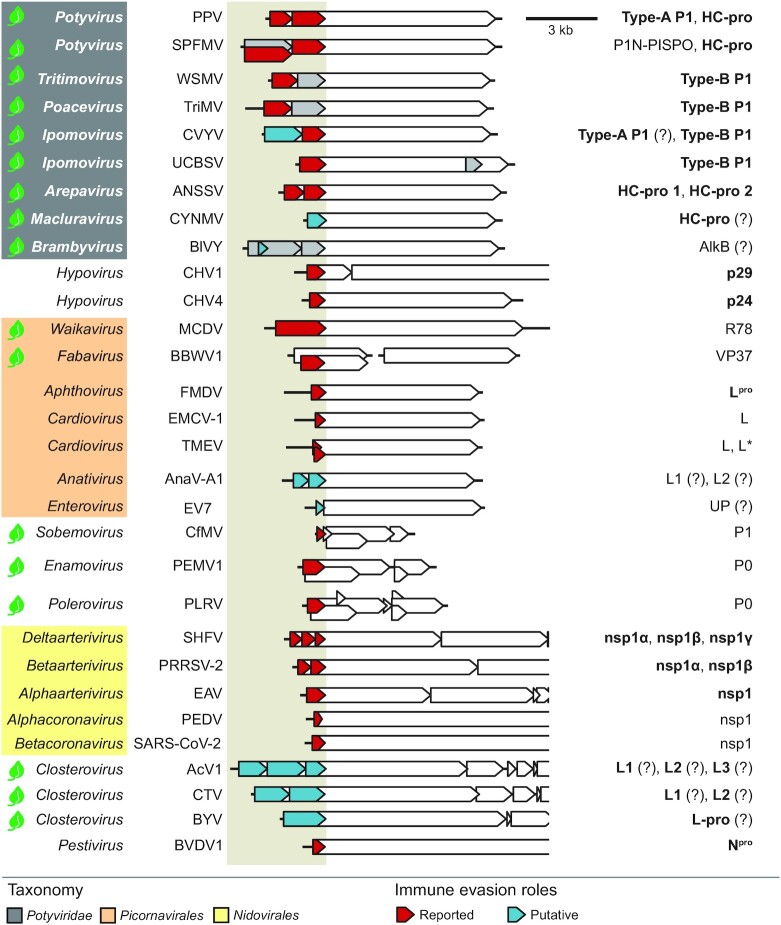
Diversity and immune evasion roles of RNA virus leader cistrons. Genomes and encoded proteins of reference RNA viruses are represented as lines and arrowed boxes, respectively. Leader and 5' cistrons with immune evasion roles are colored and their names are on the right; proteinases are in boldface. Left, genera and species are indicated. Abbreviations and functional characterization references are as follows: PPV, plum pox virus; SPFMV, sweet potato feathery mottle virus; WSMV, wheat streak mosaic virus; TriMV, *Triticum* mosaic virus; CVYV, cucumber vein yellowing virus; UCBSV, Ugandan cassava brown streak virus; ANSSV, areca palm necrotic spindle–spot virus; CYNMV, Chinese yam necrotic mosaic virus; BlVY, blackberry virus Y (see main text). CHV1, *Cryphonectria* hypovirus 1; CHV4, *Cryphonectria* hypovirus 4 (Segers *et al*. 2006, Aulia *et al*. 2021). MCDV, maize chlorotic dwarf virus; BBWV1, broad bean wilt virus 1; FMDV, foot-and-mouth disease virus; EMCV-1, encephalomyocarditis virus 1; TMEV, Theiler's murine encephalomyelitis virus; AnaV-A1, anativirus A1; EV7, echovirus 7 (Agol and Gmyl 2010, Stewart *et al*. 2017, Freundt, Drappier and Michiels 2018, Lulla *et al*. 2019, Carpino *et al*. 2020, Saiz and Martinez-Salas 2021). CfMV, cocksfoot mottle virus; PEMV1, pea enation mosaic virus 1; PLRV, potato leafroll virus (Csorba, Kontra and Burgyán 2015). SHFV, simian hemorrhagic fever virus; PRRSV-2, porcine reproductive and respiratory syndrome virus 2; EAV, equine arteritis virus; PEDV, porcine epidemic diarrhea virus; SARS-CoV-2, severe acute respiratory syndrome coronavirus 2 (Han and Yoo 2014, Lunney *et al*. 2016, Shen *et al*. 2020, Nakagawa and Makino 2021). AcV1, actinidia virus 1; CTV, citrus tristeza virus; BYV, beet yellows virus (Dolja, Kreuze and Valkonen 2006). BVDV1, bovine viral diarrhea virus 1 (Tautz, Tews and Meyers 2015). Leaf icons indicate plant viruses; virus taxonomy and accession numbers can be found in Table S3.

Potyvirid leaders are enriched in non-core modules (Fig. 2), and experimental evidence supports their roles in immune evasion and symptom development (Figs10 and 11). RNA silencing is a major antiviral mechanism of plants. *Potyvirus* HC-pro is among the best characterized silencing suppressors, with multiple roles that include direct sequestration of small RNA molecules and inhibition of RNA silencing factors (Valli *et al*. 2018). In addition to HC-pro, other potyvirid leader proteins have been implicated in evasion of antiviral RNA silencing (Fig. 10A).

Type-A P1 does not suppress RNA silencing in transient expression assays, and enhances potyviral infection in plants defective in RNA silencing (Young *et al*. 2012, Pasin, Simón-Mateo and García 2014), which suggests that the protein has additional roles independent of silencing suppression. P1 *cis*-expression strengthens the HC-pro activity, and improved translation in heterologous systems was implicated to this effect (Tena Fernández *et al*. 2013). P1 evolution for the mere enhancement of HC-pro expression appears unlikely, since optimization of the nucleotide Kozak context would be a more economic strategy.

Robust RNA silencing suppressor activity was nonetheless reported for several Type-B proteins common in *Ipomovirus*, *Poacevirus*, *Tritimovirus*, *Roymovirus* and *Brambyvirus* members. These viruses encode HC-pro with no detectable suppressor activity or silencing suppressor motifs, or lack the cistron altogether, as seen in some ipomoviruses. Type-B P1 binds short RNA molecules; this ability correlates with its silencing suppression activity (Valli, Dujovny and García 2008, Kenesi *et al*. 2017, Gupta and Tatineni 2019a). Other data show that RNA silencing suppressor activity of Type-B proteins is conferred by GW motifs that guide recognition and inhibition of the antiviral silencing component ARGONAUTE 1 (AGO1) (Giner *et al*. 2010, Kenesi *et al*. 2017). Motif requirements for silencing suppression activity has been investigated in Type-B homologs of *Ipomovirus*, *Poacevirus*, and *Tritimovirus* (Giner *et al*. 2010, Gupta and Tatineni 2019a,b, Chen *et al*. 2020). Type-B P1 proteolytic activity is not needed for silencing suppression of the ipomovirus SPMMV and the poacevirus *Triticum* mosaic virus (TriMV) (Giner *et al*. 2010, Gupta and Tatineni 2019a). The P1 cistron was expressed alone, however, and it is unclear if proteolysis is needed during infection to release mature, active silencing suppressors from polyproteins.

GW motifs involved Type-B protein activity are also present in the potyviral PISPO, and the SPFMV P1N-PISPO fusion acts as a silencing suppressor that functionally replaces HC-pro (Mingot *et al*. 2016, Untiveros *et al*. 2016). GW motifs are present in HC-pro; although not involved in silencing suppression, they are needed to recruit AGO1 for pro-viral functions (Pollari *et al*. 2020), which further highlights the leader cistron multifunctionality in host adaptation.

### Negative autoregulation of potyvirid infection for immune evasion

Diseases result from failures of cellular homeostasis (Kotas and Medzhitov 2015). Negative feedback and incoherent feedforward loops are major autocontrol mechanisms that allow biological systems to adapt to changing environment and perturbations without homeostasis loss, disease or autoimmunity. They regulate natural and engineered cellular systems, as well as phase transitions and adaptation to resource changes of bacteriophages (Pitsili, Phukan and Coll 2020, Brady *et al*. 2021, Frei and Khammash 2021, Yao *et al*. 2021). The importance of negative autoregulation in plant virus infection just starts to be appreciated.

Mechanisms have been reported in plant viruses that avoid cellular toxicity or excessive inhibition of antiviral pathways, which can trigger host damage and pathogen fitness loss (Paudel and Sanfaçon 2018, Križnik, Baebler and Gruden 2020). Promotion of RNA silencing spread was reported for tobamovirus movement protein and sobemovirus P1 (phylogenically unrelated to potyvirid P1), restriction of the silencing suppressor activity of cucumber mosaic virus 2b, geminiviral βC1 and polerovirus P0 was proposed to minimize host homeostasis perturbation (Vogler *et al*. 2008, Lacombe *et al*. 2010, Zhang *et al*. 2017, Ismayil *et al*. 2020, Watt *et al*. 2020, Clavel *et al*. 2021, Shukla *et al*. 2021).

HC-pro is a symptom determinant; its uncontrolled expression severely affects plant physiology, growth, fertility, and can trigger hypersensitive response or lethal necrosis (Pacheco *et al*. 2012, Valli *et al*. 2018). These findings suggest that tight control of proteins with strong silencing suppressor activity is desirable for optimal viral fitness, but how can it be achieved by an RNA virus that lacks transcriptional regulations? Recent data on P1 highlight a post-translational negative autoregulation that provides an evolutionary answer to the virus dilemma of counteracting defenses of the host without killing it. P1 can antagonize HC-pro, since the P1-HC-pro fusion lacks RNA silencing suppressor activity and could not sustain viral infection in hosts with unopposed antiviral immunity (Pasin, Simón-Mateo and García 2014). P1 is itself under autoinhibitory control. Several proteases display autoinhibitory domains or are synthesized as precursors that undergo structural rearrangements to activate (Hedstrom 2002, Gohara and Di Cera 2011, Trudeau *et al*. 2013). *N*-terminal deletions of PPV P1 identified a gain-of-function phenotype consistent with an autoinhibitory mechanism in which the *N* terminus negatively regulates P1 proteolysis, and self-cleavage results from autoinhibition relief by plant co-factor(s) (Pasin, Simón-Mateo and García 2014, Shan *et al*. 2018). A recent study model proposes the autoinhibited P1 self-cleavage as an immune evasion mechanism that regulates PPV replication through controlled release of the functional silencing suppressor HC-pro (Pasin *et al*. 2020). Self-controlled P1 processing kinetics would thus balance the strength of RNA silencing suppression with magnitude of phytohormone-mediated defense activation to mitigate resource burden and promote long-term viral fitness (Fig. 10B).

### Additional immune evasion roles of potyvirid non-core modules

RNA silencing and other RNA metabolic pathways contribute to plant defense against potyvirids (Li and Wang 2019, Xu *et al*. 2020). They are further interconnected with autoimmunity, hormonal, and autophagic responses to provide robust plant immunity and tolerance to viruses (Cui *et al*. 2020, Pasin *et al*. 2020, Pitzalis *et al*. 2020, Shukla *et al*. 2021).

HC-pro interacts physically with RNA turnover components and inhibits EXORIBONUCLEASE 4 (XRN4) to counteract antiviral RNA decay (Fig. 10A) (Li and Wang 2018, De *et al*. 2020). Clover yellow vein virus (ClYVV) P1 was involved in overcoming the recessive resistance conferred by eukaryotic translation initiation factor 4E in pea (Nakahara *et al*. 2010). Selective translation enhancement of viral genomes by TEV P1 has been reported, which might contribute to suppressing expression of host immune factors (Martínez and Daròs 2014). An evasion strategy of alphaviruses relies on disruption of stress granule formation by G3BP targeting mediated by peptide motifs that resemble the IxFG motif conserved in P1 *N* termini (Pasin, Simón-Mateo and García 2014, Panas *et al*. 2015, Reuper and Krenz 2021); P1 roles in stress granule processes are unknown.

Methylation impacts small RNA stability and loading in silencing complexes, and it is modulated by several silencing suppressors (Ji and Chen 2012, Csorba, Kontra and Burgyán 2015). HC-pro alters small RNA methylation through HUA ENHANCER1 methyltransferase interference and local disruption of the methionine cycle (Ji and Chen 2012, Ivanov *et al*. 2016, Del Toro *et al*. 2021). Roles of AlkB and its RNA demethylase activity in potyvirid infection are less clear (Fig. 10A). It has been suggested that regulation of RNA methylation during infection contributes to viral immune evasion by fine-tuning viral replication rates or by post-transcriptional control of host gene expression (van den Born *et al*. 2008, Zhang, Qian and Jia 2021). *N*^6^-methyladenosine amount modulation was recently proposed as a new plant antiviral mechanism that hinders long-distance viral movement (Martínez-Pérez *et al*. 2021), and recent data indicate it could be effective against potyvirids as supported by reported changes in *N*^6^-methyladenosine levels upon bymovirus infection (Zhang *et al*. 2021).

HAM1 and its ITPase activity were recently shown to be CBSV necrosis determinants (Tomlinson *et al*. 2019). Although the mechanistic details were not studied, levels of inosine triphosphate (an ITPase substrate) were shown to regulate key factors potentially involved in antiviral immunity such as the viral RdRp catalytic speed and possibly viral replication rates, as well as plant stress response activation (Dulin *et al*. 2015, Kazibwe *et al*. 2020).

Recent results indicate possible P1 roles in coordinating plant homeostasis during mixed infections, since the protein impaired activity of the crinivirus silencing suppressor P25 (Domingo-Calap *et al*. 2021).

### Expansion and immune evasion roles of RNA virus leaders

Diversification and immune evasion roles have been described for leaders or 5’ genomic cistrons of phylogenetically divergent groups of RNA viruses of plants, fungi and animals.

Plant RNA viruses of the *Sobemoviridae* family, genera *Enamovirus* and *Polerovirus* (family *Luteoviridae*), as well as *Waikavirus* and *Fabavirus* (family *Secoviridae*) belong to the picorna-like supergroup (Wolf *et al*. 2018). Cistrons encoded by their 5’ genomic portions show RNA silencing suppressor activity (Csorba, Kontra and Burgyán 2015, Sõmera, Sarmiento and Truve 2015, Stewart *et al*. 2017, Carpino *et al*. 2020). Leader proteinases of *Closteroviridae* (phylum *Kitrinoviricota*) affect pathogen virulence, superinfection exclusion, and promote viral amplification, possibly by viral replicase activation or subversion of host antiviral defenses (Dolja, Kreuze and Valkonen 2006, Atallah *et al*. 2016, Kang *et al*. 2018). Similar to potyvirids, proliferation of closterovirus leader proteases is reported. A single, a tandem, or three copies of leader proteinases are found, respectively, in genomes of beet yellows virus, citrus tristeza virus, and actinidia virus 1, among others (Fig. 11).

Fungal RNA viruses of *Hypoviridae* recruit leader cistrons to counteract antiviral immunity. RNA silencing suppressor activity was reported for the CHV1 leader protease p29, and p24 of *Cryphonectria* hypovirus 4 (CHV4; Fig. 11) (Segers *et al*. 2006, Aulia *et al*. 2021).

Among animal viruses and similar to potyvirids, picornaviruses show expansion of genomic layouts with highly divergent leaders (Fig. 11) (Gorbalenya and Lauber 2010, Zell 2018). Their leader proteins have a low level of structural and biochemical conservation, but share common biological functions in immune evasion (Agol and Gmyl 2010). Leader proteinase (L^pro^) of foot-and-mouth disease virus (FMDV; *Aphthovirus*) suppresses host cellular translation and antiviral responses by direct proteolysis of host translation factors and other RNA-binding proteins, signaling components, and conjugated ubiquitins (Saiz and Martinez-Salas 2021). Cardiovirus L, which is not a protease, antagonizes immune responses by suppressing interferon production, and can be functionally replaced by FMDV L^pro^ (Freundt, Drappier and Michiels 2018, Visser *et al*. 2020). Theiler's murine encephalomyelitis virus (TMEV; *Cardiovirus*) encodes the accessory L*, which directly targets RNase L ankyrin domains for interferon pathway inhibition and virus persistence promotion (Drappier *et al*. 2018). Murine but not human RNase L was found to be inhibited by L* (Drappier *et al*. 2018); this species-specific activity brings to mind the host-dependent activation of potyvirid Type-A P1 proteolysis (see above). The small protein UP was recently identified in the 5' region of diverse enteroviruses; UP modulates virus infection and tropism, and was suggested to participate in autophagy subversion for virus particle release (Lulla *et al*. 2019).

Leader size and domain organization vary considerably among members of the order *Nidovirales*. Arteriviruses are important veterinary disease agents; nsp1 is the first and most variable protein encoded. It is a leader proteinase, and up to three active copies are found in *Deltaarterivirus* (Vatter *et al*. 2014, Gulyaeva *et al*. 2017). Nsp1 proliferation resembles those of potyvirids encoding tandems of P1 or HC-pro (Fig. 2). Arterivirus nsp1 and its copies nsp1α, nsp1β, and nsp1γ counteract host immune defenses through interferon pathway suppression (Han and Yoo 2014, Lunney *et al*. 2016). Nsp1 of betacoronaviruses is released from polyprotein *N* termini to rapidly repress translation of cellular transcript and expression of innate immunity factors by 40S ribosomal subunit association (Nakagawa and Makino 2021). Immune evasion roles are conserved in nsp1 of alphacoronaviruses (Shen *et al*. 2020).

*Pestivirus* N^pro^ (phylum *Kitrinoviricota*) is an accessory leader proteinase that acts as an interferon pathway antagonist to prevent cell apoptosis (Tautz, Tews and Meyers 2015, Jo *et al*. 2019).

Roles in immune evasion thus appear to be a functional link that connects *Potyviridae* non-core modules to each other, as well as leader cistrons of potyvirids with those of multiple RNA viruses (Fig. 11).

## Biotech appeal of non-core modules

### Infectious clones—established tools for potyvirid biological characterization and biotechnological advances

The accessory nature identified in non-core modules warrants the use of suitable experimental systems for their biological role characterization. Full-length infectious clones are universal, indispensable tools for virus biology research and the development of experimental systems for investigating diseases (Pasin, Menzel and Daròs 2019, Kannan *et al*. 2020). They have been generated for members of *Potyvirus* (Domier *et al*. 1989), *Tritimovirus* (Choi *et al*. 1999), *Macluravirus* (Kondo and Fujita 2012), *Poacevirus* (Tatineni *et al*. 2015)*, Ipomovirus* (Pasin *et al*. 2017), *Celavirus* (Rose *et al*. 2019), *Arepavirus* (Qin *et al*. 2020), as well as for bipartite viruses of *Bymovirus* (You and Shirako 2010, Ohki, Sasaya and Maoka 2019). Homology-based cloning methods are revolutionizing the potyvirid infectious clone construction, since they are efficient and require limited viral sequence information (Desbiez *et al*. 2012, Zhao *et al*. 2020). T-DNA vectors with stabilizing features have been used for one-step assembly of potyvirid clones suitable for *Agrobacterium*-mediated delivery (Pasin *et al*. 2017, 2018). A recently developed synthetic genomics framework with plant virome capacity could streamline characterization and engineering of plant viruses with no biological material need (Pasin 2021).

### Non-core module characterization to guide plant expression vector development

**Figure 12.**
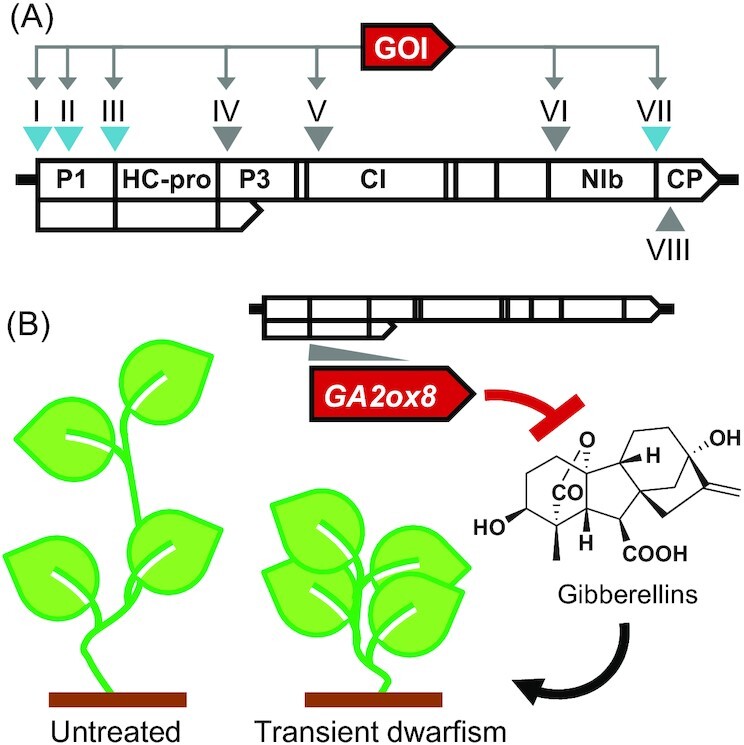
Potyvirid vectors for sequence delivery and expression in plants. **(A)** Diagram of a potyvirid genome and its insertion sites suitable for gene expression; GOI, gene of interest; site labels are as follows: I, upstream of P1; II, P1 *N* terminus; III, P1/HC-pro junction; IV, P3 *N* terminus; V, CI *N* terminus; VI, NIb *N* terminus; VII, NIb/CP junction (Rajamäki *et al*. 2005, Chen *et al*. 2007, Majer, Navarro and Daròs 2015); VIII indicates the CP *N* terminus, which has been used for heterologous peptide expression (Sánchez and Ponz 2018). Sites involved in natural gene gain events are in cyan. **(B)** Transient, potyvirid-mediated manipulation of crop traits. Right, a potyvirid vector is used to confer plant dwarfism through over-expression of a gibberellin catabolic enzyme gene (*GA2ox8*) inserted at the P1/HC-pro junction (Torti *et al*. 2021); left, growth of an untreated plant with unaltered gibberellin levels.

Virus infectious clones can be engineered and optimized as expression vectors for plant biotechnology and synthetic biology (Fig. 12) (Pasin, Menzel and Daròs 2019, Khakhar and Voytas 2021). Vectors based on potyvirids have been applied for disparate uses, ranging from production of heterologous peptides in plants, to flowering induction, gene silencing, metabolic engineering, CRISPR/Cas-targeted plant genome editing, and reprogramming of crops and their organelles (Lin *et al*. 2007, Llorente *et al*. 2020, Martí *et al*. 2020, Torti *et al*. 2021, Tuo *et al*. 2021, Uranga *et al*. 2021, Xie *et al*. 2021). Knowledge of Type-A P1 and its proteolytic activity has been instrumental in generating the first potyviral vectors (Fig. 12A). The bacterial β-glucuronidase (GUS) gene was inserted between TEV P1 and HC-pro, and the heterologous protein was released by polyprotein proteolysis mediated by P1 alone or in combination with NIa-pro (Dolja, McBride and Carrington 1992, Carrington *et al*. 1993). The same approach was used successfully in potyvirids encoding Type-B P1. GUS or fluorescent proteins were expressed using viral vectors derived from WSMV (*Tritimovirus*) (Choi *et al*. 2002, Tatineni *et al*. 2011), and TriMV (*Poacevirus)* (Tatineni *et al*. 2015). The 2A ‘self-cleaving’ peptides of FMDV or *Thosea asigna* virus were applied to engineer NIa-pro independent processing of potyvirid polyproteins (Tatineni *et al*. 2011, Pasin, Simón-Mateo and García 2014).

The ClYVV P1/HC-pro junction was engineered for co-expression of multiple heterologous proteins that were released by P1 and NIa-pro proteolysis (Masuta *et al*. 2000). More recently, ClYVV was used for plant overexpression of a gibberellin catabolic enzyme inserted between P1 and HC-pro (Fig. 12B). Infections of pea and broad bean plants with the recombinant ClYVV conferred dwarfism, an agronomically important trait (Torti *et al*. 2021). Traditional plant breeding is time- and cost-consuming, and innovative strategies are needed for accelerated and tailored crop trait manipulation (Steinwand and Ronald 2020, French *et al*. 2021). Transient, viral-mediated manipulation of plant size and other agronomic performance traits holds promise to become a new standard for fast, flexible crop reprogramming.

The NIb/CP polyprotein junction is an insertion site used for heterologous gene expression that mimics the natural HAM1 location in potyvirids (Fig. 2). Simultaneous insertions at the P1/HC-pro and NIb/CP junctions allowed production of two recombinant proteins from a single potyviral vector (Beauchemin, Bougie and Laliberté 2005). Consistent with the AlkB location found in potyvirids (Fig. 2), a new insertion site suitable for heterologous gene expression was identified within the P1 *N* terminus (Fig. 12A). It was used alone or in combination with inserts at the P1/HC-pro and NIb/CP junctions for production of up to three recombinant proteins (Rajamäki *et al*. 2005, Kelloniemi, Mäkinen and Valkonen 2008). Heterologous protein expression has been reported by gene insertion upstream of TEV P1 (Fig. 12A). This strategy allowed correct targeting of heterologous proteins to subcellular compartments (Majer, Navarro and Daròs 2015), and was used for metabolite production by potyvirus-mediated enzyme delivery to chloroplasts (Martí *et al*. 2020).

In addition to protein overexpression, heterologous sequences inserted within P1 or at the P1/HC-pro junction can trigger silencing of plant homologs (Gammelgård, Mohan and Valkonen 2007, Xie *et al*. 2021). Potyvirid vectors have been used for virus-induced gene silencing, as well as for simultaneous plant gene silencing and heterologous protein production (Gammelgård, Mohan and Valkonen 2007, Tuo *et al*. 2021, Xie *et al*. 2021).

### Non-core modules—untapped synthetic biology resources

Given their stringent specificity and orthogonality, potyvirid proteinases have been engineered for commercial purposes as well as for synthetic biology applications to control cellular functions (Chung and Lin 2020, Dyer and Weiss 2021). These proteins have been integrated into synthetic signaling pathways with designs that included induction of degron-dependent protein depletion, autoinhibition release of transcription regulators, and enzyme reconstitution through dimerization inhibition or activation (Fernandez-Rodriguez and Voigt 2016, Gao *et al*. 2018, Fink *et al*. 2019). Use of potyvirid leader proteinases in synthetic genetic circuitries has not yet been reported. Given its activation requirements and strict *cis*-cleavage activity, Type-A P1 could nonetheless be an appealing choice for biodesigns with high host specificity or biocontainment levels.

Synthetic, tight control over protein activity can be achieved by destabilizing tags, oligomerization domains, inhibitory modules, or subcellar sequestration signals (Alberstein, Guo and Kortemme 2021, Chen and Elowitz 2021). Type-A P1 was shown to undergone rapid degradation in plants and to inhibit activity of downstream fusion partners, such as HC-Pro or GUS (Verchot and Carrington 1995, Martínez and Daròs 2014, Pasin, Simón-Mateo and García 2014, Shan *et al*. 2015), and could be repurposed for conditional, fine-tuned activation of recombinant proteins.

HC-pro and other RNA silencing suppressors from plant viruses are used routinely to enhance protein yields of plant transient expression systems (Csorba, Kontra and Burgyán 2015, Sainsbury 2020); Type-B proteins and P1N-PISPO could be also useful in similar applications. In-depth characterization of potyvirid AlkB and ITPase activities may also lead to novel tools for epigenetic or metabolic engineering applications.

Virus-directed continuous evolution has been used to obtain biomolecules with improved or new functions in prokaryotic and mammalian systems (Morrison, Podracky and Liu 2020), but suitable methods are lacking for plants. Experimental studies aiming to evaluated the evolutionary fate of sequences inserted in potyvirid genomes have revealed constraints linked to the evolutionary time, as well as genome position and specific insert features (Willemsen *et al*. 2016, 2017, Willemsen and Zwart 2019). A potyviral reverse genetic system was nonetheless engineered for forced evolution of P1 proteins (Rodamilans, Casillas and García 2021).

### Leader proteinases—overlooked targets for antiviral strategies

Human viruses are targeted by proteinase inhibitor therapies to a clinically useful level (Agbowuro *et al*. 2018); yet the use of similar antiviral strategies for plant virus control is lagging. Use of protease inhibitors for potyvirus control has shown limited success so far (Gutierrez-Campos *et al*. 1999), but new promising antiviral strategies have been reported. A plant protein involved in bacterial immunity was successfully repurposed to specifically sense NIa-pro and trigger antiviral cell death (Kim *et al*. 2016). This synthetic antiviral system has been implemented in soybean, and further optimized for enhanced control of the NIa-pro-induced cell necrosis (Helm *et al*. 2019, Pottinger *et al*. 2020). Potyvirid leader proteinases are attractive antiviral targets, since P1 and HC-pro defects preclude infectivity (Kasschau and Carrington 1995, Verchot and Carrington 1995, Pasin, Simón-Mateo and García 2014, Shan *et al*. 2015). A zucchini yellow mosaic virus isolate with reduced HC-pro silencing suppressor activity has been registered since 2007 for the US market as a cross-protection agent of cucurbits (U.S. Environmental Protection Agency 2007). RNA silencing transgenic approaches that target P1 or HC-pro confer potyvirid resistance in crops (Di Nicola-Negri *et al*. 2005).

Investigation of non-core module roles in plant-potyvirid interactions recently allowed identification of new host factors and signaling pathways that could be exploited in antiviral strategies. High abscisic acid (ABA) levels were found to accumulate during infection of a PPV mutant having a truncated P1 (Fig. 10B); the finding prompted evaluation of ABA effects on infection. Defects of the cap-binding complex components ABA HYPERSENSITIVE1/CAP BINDING PROTEIN 80 (ABH1/CBP80) and CAP BINDING PROTEIN 20 (CBP20) are known to confer ABA hypersensitivity and were shown to significantly delay PPV infection (Pasin *et al*. 2020). Cap-binding complex contribution in antiviral defense was reported in other organisms, including insects and mammals (Gebhardt *et al*. 2019, Blagrove and Barribeau 2021). ABA treatments promote resistance to PPV, and possibly to other potyvirids (Alazem, Widyasari and Kim 2019, Zhang *et al*. 2019, Pasin *et al*. 2020, Chiu *et al*. 2021). Rapid catabolism, photolability, and chemical instability make ABA unsuited for agricultural purposes. Availability of synthetic ABA receptor agonists with high stability and binding affinities nonetheless paves the way for crop antiviral strategies based on chemical manipulation of ABA signaling (Hewage *et al*. 2020).

## Research outlooks and conclusions

The phylum *Pisuriviricota* includes extremely diversified RNA viruses whose radiation was proposed to be concomitant with key eukaryogenesis events (Koonin *et al*. 2008). *Potyviridae* is currently the largest family of *Riboviria* (Fig. 1A), yet thousands of novel RNA viruses await accommodation in recognized taxa (Callanan *et al*. 2020, Edgar *et al*. 2022).

Gene gain and loss, specialization, and *de novo* emergence have promoted the diversification of leader layouts of *Potyviridae* (Figs 2 and 9), as well as of divergent RNA viruses of plants and animals, e.g. closteroviruses, picornaviruses and arteriviruses (Dolja, Kreuze and Valkonen 2006, Valli, López-Moya and García 2007, Agol and Gmyl 2010, Gorbalenya and Lauber 2010, Gulyaeva *et al*. 2017, Zell 2018). Functional expansion of a polyprotein core through domain gain is hypothesized to have taken part in the evolutionary transition from plastroviruses to modern potyvirids (Lauber *et al*. 2019). We point out that evolution of potyvirid non-core domains is diverse and can potentially be traced to multiple or single acquisition events (see AlkB in *Potyvirus* and *Brambyvirus*, or the pseudo TMV-like CP in *Bymovirus*, respectively; Figs 5 and 7), recombination and retention of functionally divergent homologs (P1 tandem in *Ipomovirus*; Fig. 3), as well as emergence of a new, overlapping protein module through overprinting (PISPO in *Potyvirus*). In-depth database search and sequence analyses uncovered a putative HC-pro-like domain within *Celavirus* (Fig. 8C), as well as the presence of the ITPase fold (HAM1, a former oddity of a narrow group of potyvirids) in taxonomically divergent RNA and DNA viruses (Fig. 6).

Identification of factors that interact with potyvirid non-core proteins and elucidation of host perturbations linked to their functional alteration are indeed major research priorities for dissecting their niche adaptation roles. Complete kinetic models were described for RNA viruses that share with potyvirids similar genome replication and protein expression strategies (Zitzmann *et al*. 2020, Lopacinski *et al*. 2021). Mathematical models could provide a quantitative understanding of the complex dynamics that regulate potyvirid replication as well as host immune responses and viral counterstrategies (Pasin *et al*. 2020). Accessory genes shape cellular pangenome diversity and are enriched in plant–microbe interaction determinants (Box 1), and models have been developed to describe pangenome gene content variation (Domingo-Sananes and McInerney 2021). Can empirical data from plant-potyvirid systems contribute to theoretical frameworks for understanding cellular pangenome evolution and ecological niche adaptation?

Functional redundancy of non-core modules has allowed the establishment of relationships between potyviral HC-pro and the non-canonical silencing suppressors P1N-PISPO and Type-B P1. HC-pro counteracts RNA decay antiviral defenses and associates with RNA turnover components for infection enhancement (Li and Wang 2018, De *et al*. 2020). Are these and additional HC-pro activities performed by other non-core modules? P1, HC-pro, and HAM1 are involved in symptom development (Valli *et al*. 2018, Tomlinson *et al*. 2019, Pasin *et al*. 2020). Is there any mechanistic connection between these otherwise structurally unrelated modules? Members of *Nidovirales*, the largest known RNA viruses, have evolved proofreading replication for maintaining integrity of genomes that can reach ∼40 kb (Robson *et al*. 2020). Potyvirids are among the largest plant viruses and have unusually low mutation rates estimated to be in the range 10^−5^–10^−6^ mutations/site/generation (Sanjuán *et al*. 2010, Tromas and Elena 2010). Are functions carried out by AlkB, HAM1, or other modules conditioning the potyvirid evolution rates? Answers to these questions will assist in the better understanding contribution of the non-core proteome expansion in the *Potyviridae* evolutionary radiation and RNA virus evolution.

Finally, driven by advances in high-throughput sequencing technologies and easy access to underexplored geographical and ecological areas (Villamor *et al*. 2019, Maclot *et al*. 2020, Sommers *et al*. 2021), discovery of new potyvirids and poty-like ancestors with unusual genomic organization, atypical protein modules, and niche-optimized traits is likely to be further expanded in the near future (Lauber *et al*. 2019, Wolf *et al*. 2020). Extending efforts for potyvirid discovery and proteome functional characterization would improve understanding of non-core module roles in host adaptation evolution to eventually guide design of novel antiviral strategies and synthetic biology solutions.

**Box 1. Non-core genes in plant–microbe interactions**

Host niche adaptation is a major driving force of virus evolution (Simmonds, Aiewsakun and Katzourakis 2019), and functional characterization of the *Potyviridae* non-core modules supports their roles in symptom development and antiviral immunity evasion (main text). Pangenomes are increasingly used to represent known structural variants of cellular taxa, wherein the adaptive nature of accessory and rare genes is subject to debate (Domingo-Sananes and McInerney 2021, Coelho *et al*. 2022). Recent data nonetheless highlight the critical contribution of gene content variation in the evolution of plant-microbe dynamics and the genetic potential of holobionts (Badet and Croll 2020, Zilber-Rosenberg and Rosenberg 2021). Plant pathogenicity and adaptation factors are components of accessory genomes, lineage-specific replicons or chromosomes of bacterial and fungal species (Ma *et al*. 2010, Levy *et al*. 2017, Laflamme *et al*. 2020, Langner *et al*. 2021, Chou *et al*. 2022). Among plants, non-core genes of *Arabidopsis thaliana*, rice, rapeseed, cabbage, sunflower, and wheat are known actuators of the host–pathogen warfare (Zhao *et al*. 2018, Hübner *et al*. 2019, Van de Weyer *et al*. 2019, Bayer *et al*. 2020, Upadhyaya *et al*. 2021).

## Supplementary Material

fuac011_Supplemental_File
