# Supplementary material for: Proteome expansion in the Potyviridae evolutionary radiation
Source: FEMS Microbiol Rev. 2022 Feb 23;46(4):fuac011. doi: 10.1093/femsre/fuac011 (PMC9249622; doi:10.1093/femsre/fuac011)
Supplement: fuac011_Supplemental_File [file fuac011_supplemental_file.pdf]

SUPPORTING INFORMATION

Proteome expansion in the *Potyviridae* evolutionary radiation

Fabio Pasin[1,*], José-Antonio Daròs[2], Ioannis E. Tzanetakis[3]

[1] Instituto de Biología Molecular y Celular de Plantas (IBMCP), Consejo Superior de Investigaciones Científicas-Universitat Politècnica de València (CSIC-UPV), Valencia, Spain; School of Science, University of Padova, Padova, Italy – ORCID ID: 0000-0002-9620-4301
[2] Instituto de Biología Molecular y Celular de Plantas (IBMCP), Consejo Superior de Investigaciones Científicas-Universitat Politècnica de València (CSIC-UPV), Valencia, Spain – ORCID ID: 0000-0002-6535-2889
[3] Department of Entomology and Plant Pathology, Division of Agriculture, University of Arkansas System, Fayetteville AR, U.S.A. – ORCID ID: 0000-0002-5970-1763

*** Correspondence:** f.pasin@csic.es

SUPPORTING METHODS

To revise the diversity of *Potyviridae* proteomes comprehensively and confidently, reference isolates of recognized species were obtained from International Committee on Taxonomy of Viruses (2020); sequences with complete or near-complete genomic sequences were analyzed (n = 185). Protein domains were identified by BLAST search, or by sensitive homology scan using domain signatures and HMM profiles from the Prosite, Pfam or InterPro databases (Sigrist *et al.* 2013; Blum *et al.* 2021; Mistry *et al.* 2021). Enzyme domain signatures of NIa-pro, CI, and NIb were used for the core proteome mapping. NIa-pro could not be detected in the divergent celery latent virus (CeLV) by Pfam analyses (Gibbs *et al.* 2020); however, it is identified by the InterPro profile IPR009003 in all potyvirid polyproteins, including those of CeLV (AZJ53460.1), and the related *Striga* potyvirus B (QVG60634.1). Domain distribution within virus taxa was retrieved from InterPro, and integrated by BLAST searches against the NCBI database (May 2021). A final *Potyviridae* inventory of the non-core domains discussed is given in Table S2.

Protein sequences were aligned using MAFFT with the G-INS-i refinement method (Katoh, Rozewicki and Yamada 2019). Alignments was manually trimmed, and phylogeny was inferred using IQ-TREE (Trifinopoulos *et al.* 2016), with ModelFinder together with ultrafast bootstrap (×1000) and SH-aLRT supports (×1000); the final trees were visualized using iTOL (Letunic and Bork 2021).

SUPPORTING TABLES

Table S1. Species and genus abundance in families of the *Riboviria* realm

Table S2. Non-core module inventory of *Potyviridae* proteomes

Table S3. Taxonomy and accession numbers of the viruses depicted in Figure 11

SUPPORTING FIGURE LEGENDS

Figure S1. Alignment of P1 protease domains. Domains were identified and protein sequences were aligned as described in Supporting Methods; accession numbers are shown or given in Table S2.

Figure S2. Alignment of AlkB domains. Domains were identified and protein sequences were aligned as described in Supporting Methods; accession numbers are shown or given in Table S2.

Figure S3. Alignment of TMV-like CP domains. Domains were identified and protein sequences were aligned as described in Supporting Methods; accession numbers are shown or given in Table S2.

Figure S4. Alignment of HC-pro protease domains. Domains were identified and protein sequences were aligned as described in Supporting Methods; accession numbers are shown or given in Table S2.

Figure S5. Alignment of RdRp domains. Domains were identified and protein sequences were aligned as described in Supporting Methods; accession numbers are shown or given in Table S2.

SUPPORTING REFERENCES

Blum M, Chang H-Y, Chuguransky S *et al.* The InterPro protein families and domains database: 20 years on. *Nucleic Acids Research* 2021;**49**:D344–54.

Gibbs AJ, Hajizadeh M, Ohshima K *et al.* The potyviruses: an evolutionary synthesis is emerging. *Viruses* 2020;**12**:132.

International Committee on Taxonomy of Viruses 2020. ICTV master species list 2019.v1 (MSL #35). Last accessed June 2021, <https://talk.ictvonline.org/files/master-species-lists/m/msl/9601>.

Katoh K, Rozewicki J, Yamada KD. MAFFT online service: multiple sequence alignment, interactive sequence choice and visualization. *Brief Bioinform* 2019;**20**:1160–6.

Letunic I, Bork P. Interactive Tree Of Life (iTOL) v5: an online tool for phylogenetic tree display and annotation. *Nucleic Acids Res* 2021:gkab301.

Mistry J, Chuguransky S, Williams L *et al.* Pfam: the protein families database in 2021. *Nucleic Acids Research* 2021;**49**:D412–9.

Sigrist CJA, de Castro E, Cerutti L *et al.* New and continuing developments at PROSITE. *Nucleic Acids Res* 2013;**41**:D344-347.

Trifinopoulos J, Nguyen L-T, von Haeseler A *et al.* W-IQ-TREE: a fast online phylogenetic tool for maximum likelihood analysis. *Nucleic Acids Res* 2016;**44**:W232–5.

Table S1. Species and genus abundance in families of the *Riboviria* realm

Family[(a)]	Genera[(a)]	Species[(a)]
Potyviridae	12	228
Rhabdoviridae	30	191
Picornaviridae	63	147
Phenuiviridae	19	117
Betaflexiviridae	13	108
Reoviridae	15	97
Peribunyaviridae	4	97
Flaviviridae	4	89
Secoviridae	8	86
Caulimoviridae	10	85
Paramyxoviridae	17	78
Tombusviridae	16	76
Retroviridae	11	68
Partitiviridae	5	60
Virgaviridae	7	59
Alphaflexiviridae	7	56
Closteroviridae	4	56
Luteoviridae	3	51
Arenaviridae	4	50
Hantaviridae	7	48
Coronaviridae	5	46
Tymoviridae	3	41
Bromoviridae	6	36
Pseudoviridae	3	34
Endornaviridae	2	31
Metaviridae	2	31
Togaviridae	1	31
Chuviridae	1	30
Totiviridae	5	28
Tospoviridae	1	26
Chrysoviridae	2	25
Arteriviridae	13	23
Astroviridae	2	22
Marnaviridae	7	20
Solemoviridae	2	20
Hepadnaviridae	5	18
Nairoviridae	3	17
Tobaniviridae	8	15
Dicistroviridae	3	15
Iflaviridae	1	15
Polycipiviridae	3	14
Caliciviridae	11	13
Phasmaviridae	5	13
Nyamiviridae	6	12
Birnaviridae	7	11
Filoviridae	6	11
Bornaviridae	3	11
Belpaoviridae	1	11
Fimoviridae	1	11

Botourmiaviridae	4	10
Alphatetraviridae	2	10
Amalgaviridae	2	10
Mesoniviridae	1	10
Polymycoviridae	1	10
Orthomyxoviridae	7	9
Mymonaviridae	2	9
Nodaviridae	2	9
Artoviridae	2	8
Qinviridae	1	8
Aspiviridae	1	7
Cystoviridae	1	7
Xinmoviridae	1	7
Lispiviridae	1	6
Kitaviridae	3	5
Hepeviridae	2	5
Pneumoviridae	2	5
Mitoviridae	1	5
Leviviridae	2	4
Mayoviridae	2	4
Benyviridae	1	4
Hypoviridae	1	4
Euroniviridae	2	3
Deltaflexiviridae	1	3
Picobirnaviridae	1	3
Roniviridae	1	3
Medioniviridae	2	2
Solinviviridae	2	2
Narnaviridae	1	2
Permutotetraviridae	1	2
Sinhaliviridae	1	2
Yueviridae	1	2
Abyssoviridae	1	1
Alvernaviridae	1	1
Amnoonviridae	1	1
Barnaviridae	1	1
Carmotetraviridae	1	1
Cremegaviridae	1	1
Cruliviridae	1	1
Gammaflexiviridae	1	1
Gresnaviridae	1	1
Leishbuviridae	1	1
Matonaviridae	1	1
Megabirnaviridae	1	1
Mononiviridae	1	1
Mypoviridae	1	1
Nanghoshaviridae	1	1
Nanhypoviridae	1	1
Olifoviridae	1	1
Quadriviridae	1	1
Sarthroviridae	1	1

Sunviridae	1	1
Wupedeviridae	1	1

[a] Data from International Committee on Taxonomy of Viruses, 2020. ICTV master species list 2019.v1 (MSL #35). Last accessed June 2021, <https://talk.ictvonline.org/files/master-species-lists/m/msl/9601>.

Table S2. Non-core module inventory of *Potyviridae* proteomes

Family	Genus	Species	Virus_name_abbreviation	Genome_accession	Polyprotein_accession	P1_Type_A	P1_Type_B	PISPO	DUF3725	AlkB	HAM1	TMV-like_CP	HC-pro	Core[(a)]
Potyviridae	*Arepavirus*	*Areca palm necrotic ringspot virus*	ANRSV	MH395371	AXS77503.1	0	0	0	0	0	0	0	2	1
Potyviridae	*Arepavirus*	*Areca palm necrotic spindle-spot virus*	ANSSV	MH330686	AXS76840.1	0	0	0	0	0	0	0	2	1
Potyviridae	*Bevemovirus*	*Bellflower veinal mottle virus*	BVMoV	KY491536	ARM19740.1	0	0	0	0	0	0	0	1	1
Potyviridae	*Brambyvirus*	*Blackberry virus Y*	BlVY	AY994084	AAX87001.1	0	1	0	0	1	0	0	1	1
Potyviridae	*Bymovirus*	*Barley mild mosaic virus*	BaMMV	X90904, Y10973	CAA62412.1, CAA71869.1	0	0	0	0	0	0	1	1	1
Potyviridae	*Bymovirus*	*Barley yellow mosaic virus*	BaYMV	AJ132268, AJ132269	CAA10637.1, CAA10638.1	0	0	0	0	0	0	1	1	1
Potyviridae	*Bymovirus*	*Oat mosaic virus*	OMV	AJ306719, AJ306718	CAC84676.1, CAC84680.1	0	0	0	0	0	0	0	1	1
Potyviridae	*Bymovirus*	*Rice necrosis mosaic virus*	RNMV	LC055681, LC060925	BAT23038.1, BAT23039.1	0	0	0	0	0	0	1	1	1
Potyviridae	*Bymovirus*	*Wheat spindle streak mosaic virus*	WSSMV	MN046367, MN046369	QGT40987.1, QGT40989.1	0	0	0	0	0	0	1	1	1
Potyviridae	*Bymovirus*	*Wheat yellow mosaic virus*	WYMV	D86634, D86635	BAA28768.1, BAA28769.1	0	0	0	0	0	0	1	1	1
Potyviridae	*Celavirus*	*Celery latent virus*	CeLV	MH932227	AZJ53460.1	0	0	0	0	0	0	0	0	1
Potyviridae	*Ipomovirus*	*Cassava brown streak virus*	CBSV	GU563327	ADR73022.1	0	1	0	0	0	1	0	0	1
Potyviridae	*Ipomovirus*	*Coccinia mottle virus*	CocMoV	KU935732	AOC84052.1	1	1	0	1	0	0	0	0	1
Potyviridae	*Ipomovirus*	*Cucumber vein yellowing virus*	CVYV	AY578085	AAT66639.1	1	1	0	1	0	0	0	0	1
Potyviridae	*Ipomovirus*	*Squash vein yellowing virus*	SqVYV	EU259611	ABY86626.1	1	1	0	0	0	0	0	0	1
Potyviridae	*Ipomovirus*	*Sweet potato mild mottle virus*	SPMMV	Z73124	CAA97466.1	0	1	0	0	0	0	0	1	1
Potyviridae	*Ipomovirus*	*Tomato mild mottle virus*	TMMoV	HE600072	CCD57807.1	0	1	0	0	0	0	0	1	1
Potyviridae	*Ipomovirus*	*Ugandan cassava brown streak virus*	UCBSV	FJ185044	ACN50007.1	0	1	0	0	0	1	0	0	1
Potyviridae	*Macluravirus*	*Alpinia oxyphylla mosaic virus*	AloMV	MG978107	AWO77093.1	0	0	0	0	0	0	0	1	1
Potyviridae	*Macluravirus*	*Artichoke latent virus*	ArLV	KP405232	AJW83674.1	0	0	0	0	0	0	0	1	1
Potyviridae	*Macluravirus*	*Broad-leafed dock virus A*	BDVA	KU053507	AMN08808.1	0	0	0	0	0	0	0	1	1
Potyviridae	*Macluravirus*	*Cardamom mosaic virus*	CdMV	MF622947	ATO88014.1	0	0	0	0	0	0	0	1	1
Potyviridae	*Macluravirus*	*Chinese yam necrotic mosaic virus*	CYNMV	AB710145	BAM36463.1	0	0	0	0	0	0	0	1	1
Potyviridae	*Macluravirus*	*Narcissus latent virus*	NLV	KX979913	ATO58458.1	0	0	0	0	0	0	0	1	1
Potyviridae	*Macluravirus*	*Yam chlorotic mosaic virus*	YCMV	KT724961	AMQ75908.1	0	0	0	0	0	0	0	1	1
Potyviridae	*Macluravirus*	*Yam chlorotic necrosis virus*	YCNV	MG755240	AWH61232.1	0	0	0	0	0	0	0	1	1
Potyviridae	*Poacevirus*	*Caladenia virus A*	CalVA	JX156425	AFQ95549.1	0	1	0	0	0	0	0	1	1
Potyviridae	*Poacevirus*	*Sugarcane streak mosaic virus*	SCSMV	GQ388116	ADE34528.1	0	1	0	0	0	0	0	1	1
Potyviridae	*Poacevirus*	*Triticum mosaic virus*	TriMV	FJ263671	ABO41208.2	0	1	0	0	0	0	0	1	1
Potyviridae	*Potyvirus*	*African eggplant mosaic virus*	AEMV	MF997470	ATY46581.1	1	0	0	0	0	0	0	1	1
Potyviridae	*Potyvirus*	*Algerian watermelon mosaic virus*	AWMV	EU410442	ACB88930.1	1	0	0	0	0	0	0	1	1
Potyviridae	*Potyvirus*	*Alstroemeria mosaic virus*	AlMV	MK440140	QDA01852.2	1	0	0	0	0	0	0	1	1
Potyviridae	*Potyvirus*	*Amaranthus leaf mottle virus*	AmLMV	MN709786	QHO60634.1	1	0	0	0	0	0	0	1	1
Potyviridae	*Potyvirus*	*Apium virus Y*	ApVY	HM363516	ADT71770.1	1	0	0	0	0	0	0	1	1
Potyviridae	*Potyvirus*	*Arracacha mottle virus*	AMoV	DQ925486	ABI97034.2	1	0	0	0	0	0	0	1	1
Potyviridae	*Potyvirus*	*Asparagus virus 1*	AV1	KJ830760	AIY55492.1	1	0	0	0	0	0	0	1	1
Potyviridae	*Potyvirus*	*Banana bract mosaic virus*	BBrMV	DQ851496	ABI34615.1	1	0	0	0	0	0	0	1	1
Potyviridae	*Potyvirus*	*Barbacena virus Y*	BarVY	KU685505	AOC37868.1	1	0	0	0	0	0	0	1	1
Potyviridae	*Potyvirus*	*Basella rugose mosaic virus*	BaRMV	DQ821938	ABH10134.1	1	0	0	0	0	0	0	1	1
Potyviridae	*Potyvirus*	*Bean common mosaic necrosis virus*	BCMNV	U19287	AAB02170.1	1	0	0	0	0	0	0	1	1
Potyviridae	*Potyvirus*	*Bean common mosaic virus*	BlCMV	AJ312437	CAC86160.1	1	0	0	0	0	0	0	1	1
Potyviridae	*Potyvirus*	*Bean yellow mosaic virus*	BYMV	D83749	BAA12099.1	1	0	0	0	0	0	0	1	1
Potyviridae	*Potyvirus*	*Beet mosaic virus*	BtMV	AY206394	AAP41071.1	1	0	0	0	0	0	0	1	1
Potyviridae	*Potyvirus*	*Bidens mosaic virus*	BiMV	KF649336	AHB50526.1	1	0	0	0	0	0	0	1	1
Potyviridae	*Potyvirus*	*Bidens mottle virus*	BiMoV	AF538686	AAO37457.2	1	0	0	0	0	0	0	1	1
Potyviridae	*Potyvirus*	*Blue squill virus A*	BSVA	JQ807999	AFV61776.1	1	0	0	0	0	0	0	1	1
Potyviridae	*Potyvirus*	*Brugmansia mosaic virus*	BruMV	JX867236	AGC08387.1	1	0	0	0	0	0	0	1	1
Potyviridae	*Potyvirus*	*Brugmansia suaveolens mottle virus*	BsMoV	AB551370	BAJ19142.1	1	0	0	0	0	0	0	1	1
Potyviridae	*Potyvirus*	*Calla lily latent virus*	CLLV	EF105299	ABO77135.1	1	0	0	0	0	0	0	1	1
Potyviridae	*Potyvirus*	*Callistephus mottle virus*	CalMoV	KX013584	ANY95172.1	1	0	0	0	0	0	0	1	1
Potyviridae	*Potyvirus*	*Canna yellow streak virus*	CaYSV	GQ421689	ACV84257.2	1	0	0	0	0	0	0	1	1
Potyviridae	*Potyvirus*	*Carrot thin leaf virus*	CTLV	JX156434	AGH25888.1	1	0	0	0	0	0	0	1	1
Potyviridae	*Potyvirus*	*Carrot virus Y*	CarVY	LC511903	BBP49732.1	1	0	0	0	0	0	0	1	1
Potyviridae	*Potyvirus*	*Catharanthus mosaic virus*	CatMV	KP343681	AIU97943.2	1	0	0	0	0	0	0	1	1
Potyviridae	*Potyvirus*	*Celery mosaic virus*	CeMV	HQ676607	AEB00568.1	1	0	0	0	0	0	0	1	1
Potyviridae	*Potyvirus*	*Chilli ringspot virus*	CRSV	JN008909	AEP37329.1	1	0	0	0	0	0	0	1	1
Potyviridae	*Potyvirus*	*Chilli veinal mottle virus*	ChiVMV	AJ237843	CAB43195.2	1	0	0	0	0	0	0	1	1
Potyviridae	*Potyvirus*	*Clover yellow vein virus*	ClYVV	AB011819	BAA25147.1	1	0	0	0	0	0	0	1	1
Potyviridae	*Potyvirus*	*Cocksfoot streak virus*	CSV	AF499738	AAM19343.1	1	0	0	0	0	0	0	1	1
Potyviridae	*Potyvirus*	*Colombian datura virus*	CDV	JQ801448	AGA93117.1	1	0	0	0	0	0	0	1	1

Potyviridae	*Potyvirus*	*Cowpea aphid-borne mosaic virus*	CABMV	AF348210	AAL83896.1	1	0	0	0	0	0	0	1	1
Potyviridae	*Potyvirus*	*Cucurbit vein banding virus*	CVBV	KY657266	ASB15793.1	1	0	0	0	0	0	0	1	1
Potyviridae	*Potyvirus*	*Cyrtanthus elatus virus A*	CEVA	JQ723475	AFJ92921.1	1	0	0	0	0	0	0	1	1
Potyviridae	*Potyvirus*	*Daphne mosaic virus*	DapMV	DQ299908	ABC25565.1	1	0	0	0	0	0	0	1	1
Potyviridae	*Potyvirus*	*Daphne virus Y*	DVY	KU556609	AMR93994.1	1	0	0	0	0	0	0	1	1
Potyviridae	*Potyvirus*	*Dasheen mosaic virus*	DsMV	AJ298033	CAC83052.1	1	0	0	0	0	0	0	1	1
Potyviridae	*Potyvirus*	*Dendrobium chlorotic mosaic virus*	DeCMV	MK241979	QBS16347.1	1	0	0	0	0	0	0	1	1
Potyviridae	*Potyvirus*	*Dioscorea mosaic virus*	DMV	MH206616	AZB50213.1	1	0	0	0	0	0	0	1	1
Potyviridae	*Potyvirus*	*Donkey orchid virus A*	DOVA	JX156422	AFQ95553.1	1	0	0	0	0	0	0	1	1
Potyviridae	*Potyvirus*	*East Asian Passiflora distortion virus*	EAPDV	LC379162	BBD74024.1	1	0	0	0	0	0	0	1	1
Potyviridae	*Potyvirus*	*East Asian Passiflora virus*	EAPV	AB246773	BAE78404.1	1	0	0	0	0	0	0	1	1
Potyviridae	*Potyvirus*	*Endive necrotic mosaic virus*	ENMV	KU941946	ARF07717.1	1	0	0	0	1	0	0	1	1
Potyviridae	*Potyvirus*	*Euphorbia ringspot virus*	EuRSV	KX355613	AOT35732.1	1	0	0	0	0	1	0	1	1
Potyviridae	*Potyvirus*	*Freesia mosaic virus*	FreMV	FM206346	CAR58104.1	1	0	0	0	0	0	0	1	1
Potyviridae	*Potyvirus*	*Fritillary virus Y*	FVY	AM039800	CAJ01678.1	1	0	0	0	0	0	0	1	1
Potyviridae	*Potyvirus*	*Gloriosa stripe mosaic virus*	GSMV	EF427894	ABR88099.1	1	0	0	0	0	0	0	1	1
Potyviridae	*Potyvirus*	*Gomphocarpus mosaic virus*	GoMV	LC228573	BBD33993.1	1	0	0	0	0	0	0	1	1
Potyviridae	*Potyvirus*	*Habenaria mosaic virus*	HaMV	AB818538	BAN63167.1	1	0	0	0	0	0	0	1	1
Potyviridae	*Potyvirus*	*Hardenbergia mosaic virus*	HarMV	HQ161081	AEB00584.1	1	0	0	0	0	0	0	1	1
Potyviridae	*Potyvirus*	*Henbane mosaic virus*	HMV	MH779476	AZL49328.1	1	0	0	0	0	0	0	1	1
Potyviridae	*Potyvirus*	*Hippeastrum mosaic virus*	HiMV	JQ395040	AFJ92905.1	1	0	0	0	0	0	0	1	1
Potyviridae	*Potyvirus*	*Hyacinth mosaic virus*	HyaMV	KY828925	ASC55665.2	1	0	0	0	0	0	0	1	1
Potyviridae	*Potyvirus*	*Impatiens flower break virus*	IFBV	KU981084	ANA48365.1	1	0	0	0	0	0	0	1	1
Potyviridae	*Potyvirus*	*Iris mild mosaic virus*	IMMV	MH886513	QDC21205.1	1	0	0	0	0	0	0	1	1
Potyviridae	*Potyvirus*	*Iris severe mosaic virus*	ISMV	KT692938	ALX81666.1	1	0	0	0	0	0	0	1	1
Potyviridae	*Potyvirus*	*Japanese yam mosaic virus*	JYMV	AB027007	BAA86288.1	1	0	0	0	0	0	0	1	1
Potyviridae	*Potyvirus*	*Jasmine virus T*	JVT	KT222674	ALW54844.1	1	0	0	0	0	0	0	1	1
Potyviridae	*Potyvirus*	*Johnsongrass mosaic virus*	JGMV	Z26920	CAA81549.1	1	0	0	0	0	0	0	1	1
Potyviridae	*Potyvirus*	*Kalanchoe mosaic virus*	KMV	KY385304	APX54983.1	1	0	0	0	0	0	0	1	1
Potyviridae	*Potyvirus*	*Keunjorong mosaic virus*	KjMV	JF838187	AER59752.1	1	0	0	0	0	0	0	1	1
Potyviridae	*Potyvirus*	*Konjac mosaic virus*	KoMV	AB219545	BAE87001.1	1	0	0	0	0	0	0	1	1
Potyviridae	*Potyvirus*	*Leek yellow stripe virus*	LYSV	AJ307057	CAC85226.1	1	0	0	0	0	0	0	1	1
Potyviridae	*Potyvirus*	*Lettuce Italian necrotic virus*	LINV	KP769852	AKU47816.1	1	0	0	0	0	0	0	1	1
Potyviridae	*Potyvirus*	*Lettuce mosaic virus*	LMV	X97705	CAA66281.1	1	0	0	0	0	0	0	1	1
Potyviridae	*Potyvirus*	*Lily mottle virus*	LMoV	AJ564636	CAD92110.1	1	0	0	0	0	0	0	1	1
Potyviridae	*Potyvirus*	*Lily virus Y*	LVY	MF543013	AVQ05260.1	1	0	0	0	0	0	0	1	1
Potyviridae	*Potyvirus*	*Lupinus mosaic virus*	LuMV	EU847625	ACJ31798.2	1	0	0	0	0	0	0	1	1
Potyviridae	*Potyvirus*	*Maize dwarf mosaic virus*	MDMV	AJ001691	CAA04929.1	1	0	0	0	0	0	0	1	1
Potyviridae	*Potyvirus*	*Malva vein clearing virus*	MVCV	MN116683	QIA62007.1	1	0	0	0	0	0	0	1	1
Potyviridae	*Potyvirus*	*Mashua virus Y*	MasVY	MH680824	AXK90538.1	1	0	0	0	0	0	0	1	1
Potyviridae	*Potyvirus*	*Mediterranean ruda virus*	MeRV	MF953305	AUD37401.1	1	0	0	0	0	0	0	1	1
Potyviridae	*Potyvirus*	*Moroccan watermelon mosaic virus*	MWMV	EF579955	ABU93572.1	1	0	0	0	0	0	0	1	1
Potyviridae	*Potyvirus*	*Narcissus degeneration virus*	NDV	AM182028	CAJ57715.1	1	0	0	0	0	0	0	1	1
Potyviridae	*Potyvirus*	*Narcissus late season yellows virus*	NLSYV	KC691259	AHB52332.1	1	0	0	0	0	0	0	1	1
Potyviridae	*Potyvirus*	*Narcissus yellow stripe virus*	NYSV	AM158908	CAJ43612.1	1	0	0	0	0	0	0	1	1
Potyviridae	*Potyvirus*	*Nerine yellow stripe virus*	NeYSV	MT396083	QOD42425.1	1	0	0	0	0	0	0	1	1
Potyviridae	*Potyvirus*	*Onion yellow dwarf virus*	OYDV	AJ510223	CAD53318.1	1	0	0	0	0	0	0	1	1
Potyviridae	*Potyvirus*	*Ornithogalum mosaic virus*	OrMV	JQ807997	AFV61775.1	1	0	0	0	0	0	0	1	1
Potyviridae	*Potyvirus*	*Ornithogalum virus 3*	OrV3	MW035311	QRG34965.1	1	0	0	0	0	0	0	1	1
Potyviridae	*Potyvirus*	*Panax virus Y*	PanVY	GQ916624	ADI58756.1	1	0	0	0	0	0	0	1	1
Potyviridae	*Potyvirus*	*Papaya leaf distortion mosaic virus*	PLDMV	BD171712	NP_870995.1	1	0	0	0	0	0	0	1	1
Potyviridae	*Potyvirus*	*Papaya ringspot virus*	PRSV	X67673	CAA47905.1	1	0	0	1	0	0	0	1	1
Potyviridae	*Potyvirus*	*Paris mosaic necrosis virus*	PMNV	MF509898	AUB51246.1	1	0	0	0	0	0	0	1	1
Potyviridae	*Potyvirus*	*Passion fruit woodiness virus*	PWV	HQ122652	ADR10439.1	1	0	0	0	0	0	0	1	1
Potyviridae	*Potyvirus*	*Pea seed-borne mosaic virus*	PSbMV	D10930	BAA01726.1	1	0	0	0	0	0	0	1	1
Potyviridae	*Potyvirus*	*Peanut mottle virus*	PeMoV	AF023848	AAB94595.1	1	0	0	0	0	0	0	1	1
Potyviridae	*Potyvirus*	*Pecan mosaic-associated virus*	PMaV	KT633868	ANH79182.1	1	0	0	0	0	0	0	1	1
Potyviridae	*Potyvirus*	*Pennisetum mosaic virus*	PenMV	AY642590	AAV48572.1	1	0	0	0	0	0	0	1	1
Potyviridae	*Potyvirus*	*Pepper mottle virus*	PepMoV	M96425	AAA46903.1	1	0	0	0	0	0	0	1	1
Potyviridae	*Potyvirus*	*Pepper severe mosaic virus*	PepSMV	AM181350	CAJ57401.1	1	0	0	0	0	0	0	1	1
Potyviridae	*Potyvirus*	*Pepper veinal mottle virus*	PVMV	DQ645484	ABG56784.1	1	0	0	0	0	0	0	1	1
Potyviridae	*Potyvirus*	*Pepper yellow mosaic virus*	PepYMV	AB541985	BAJ10980.1	1	0	0	0	0	0	0	1	1
Potyviridae	*Potyvirus*	*Peru tomato mosaic virus*	PTV	AJ437280	CAD24793.1	1	0	0	0	0	0	0	1	1

Potyviridae	*Potyvirus*	*Platycodon mild mottle virus*	PlaMMV	MH779625	AYA60486.1	1	0	0	0	0	0	0	1	1
Potyviridae	*Potyvirus*	*Plum pox virus*	PPV	AJ243957	CAB51641.1	1	0	0	0	0	0	0	1	1
Potyviridae	*Potyvirus*	*Pokeweed mosaic virus*	PkMV	JX291161	AFS28881.1	1	0	0	0	0	0	0	1	1
Potyviridae	*Potyvirus*	*Potato virus A*	PVA	AJ296311	CAC17411.1	1	0	0	0	0	0	0	1	1
Potyviridae	*Potyvirus*	*Potato virus V*	PVV	AJ243766	CAB75857.2	1	0	0	0	0	0	0	1	1
Potyviridae	*Potyvirus*	*Potato virus Y*	PVY	U09509	AAB50573.1	1	0	0	0	0	0	0	1	1
Potyviridae	*Potyvirus*	*Potato yellow blotch virus*	PYBV	JX294310	AFS28882.1	1	0	0	0	0	0	0	1	1
Potyviridae	*Potyvirus*	*Saffron latent virus*	SaLV	KY562565	AUP47462.1	1	0	0	0	0	0	0	1	1
Potyviridae	*Potyvirus*	*Scallion mosaic virus*	ScaMV	AJ316084	CAC87085.1	1	0	0	0	0	0	0	1	1
Potyviridae	*Potyvirus*	*Shallot yellow stripe virus*	SYSV	AJ865076	CAI23782.1	1	0	0	0	0	0	0	1	1
Potyviridae	*Potyvirus*	*Sorghum mosaic virus*	SrMV	AJ310197	CAC84437.1	1	0	0	0	0	0	0	1	1
Potyviridae	*Potyvirus*	*Soybean mosaic virus*	SMV	D00507	BAA00398.2	1	0	0	0	0	0	0	1	1
Potyviridae	*Potyvirus*	*Sudan watermelon mosaic virus*	SuWMV	KY623505	ASK09431.1	1	0	0	1	0	0	0	1	1
Potyviridae	*Potyvirus*	*Sugarcane mosaic virus*	SCMV	AJ297628	CAC82225.1	1	0	0	0	0	0	0	1	1
Potyviridae	*Potyvirus*	*Sunflower chlorotic mottle virus*	SCMoV	GU181199	ADF31931.1	1	0	0	0	0	0	0	1	1
Potyviridae	*Potyvirus*	*Sunflower mild mosaic virus*	SMMV	JQ350738	AFU72533.1	1	0	0	0	0	0	0	1	1
Potyviridae	*Potyvirus*	*Sunflower ring blotch virus*	SuRBV	KX856009	AQU42721.1	1	0	0	0	0	0	0	1	1
Potyviridae	*Potyvirus*	*Sweet potato feathery mottle virus*	SPFMV	D86371	BAA22702.1	1	0	1	0	0	0	0	1	1
Potyviridae	*Potyvirus*	*Sweet potato latent virus*	SPLV	KC443039	AGH25932.1	1	0	0	0	0	0	0	1	1
Potyviridae	*Potyvirus*	*Sweet potato virus 2*	SPV2	JN613807	AFJ68041.1	1	0	1	0	0	0	0	1	1
Potyviridae	*Potyvirus*	*Sweet potato virus C*	SPVC	GU207957	ADQ74918.1	1	0	1	0	0	0	0	1	1
Potyviridae	*Potyvirus*	*Sweet potato virus G*	SPVG	JQ824374	AFM30943.1	1	0	1	0	0	0	0	1	1
Potyviridae	*Potyvirus*	*Tamarillo leaf malformation virus*	TLMV	KM523548	AJO62057.1	1	0	0	0	0	0	0	1	1
Potyviridae	*Potyvirus*	*Telosma mosaic virus*	TelMV	DQ851493	ABI34612.1	1	0	0	0	0	0	0	1	1
Potyviridae	*Potyvirus*	*Thunberg fritillary mosaic virus*	TFMV	AJ851866	CAH65461.1	1	0	0	0	0	0	0	1	1
Potyviridae	*Potyvirus*	*Tobacco etch virus*	TEV	M11458	AAA47909.1	1	0	0	0	0	0	0	1	1
Potyviridae	*Potyvirus*	*Tobacco mosqueado virus*	TMosqV	KT834407	AMC38503.1	1	0	0	0	0	0	0	1	1
Potyviridae	*Potyvirus*	*Tobacco vein banding mosaic virus*	TVBMV	EF219408	ABP87907.1	1	0	0	0	0	0	0	1	1
Potyviridae	*Potyvirus*	*Tobacco vein mottling virus*	TVMV	X04083	CAA27720.1	1	0	0	0	0	0	0	1	1
Potyviridae	*Potyvirus*	*Tomato necrotic stunt virus*	TNSV	JQ314463	AFI25344.1	1	0	0	0	0	0	0	1	1
Potyviridae	*Potyvirus*	*Tulip breaking virus*	TBV	MH886517	QDC21209.1	1	0	0	0	0	0	0	1	1
Potyviridae	*Potyvirus*	*Turnip mosaic virus*	TuMV	AF169561	AAF89676.2	1	0	0	0	0	0	0	1	1
Potyviridae	*Potyvirus*	*Vanilla distortion mosaic virus*	VDMV	KF906523	AHU88030.1	1	0	0	0	0	0	0	1	1
Potyviridae	*Potyvirus*	*Verbena virus Y*	VVY	EU564817	ACB69755.1	1	0	0	0	0	0	0	1	1
Potyviridae	*Potyvirus*	*Watermelon mosaic virus*	WMV	AY437609	AAR99062.1	1	0	0	0	0	0	0	1	1
Potyviridae	*Potyvirus*	*Wild melon banding virus*	WMVBV	KY623506	ASK09432.1	1	0	0	1	0	0	0	1	1
Potyviridae	*Potyvirus*	*Wild onion symptomless virus*	WoSV	LC159494	BAV17838.1	1	0	0	0	0	0	0	1	1
Potyviridae	*Potyvirus*	*Wild potato mosaic virus*	WPMV	AJ437279	CAD24792.1	1	0	0	0	0	0	0	1	1
Potyviridae	*Potyvirus*	*Wild tomato mosaic virus*	WTMV	DQ851495	ABI34614.1	1	0	0	0	0	0	0	1	1
Potyviridae	*Potyvirus*	*Wisteria vein mosaic virus*	WVMV	AY656816	AAV68594.1	1	0	0	0	0	0	0	1	1
Potyviridae	*Potyvirus*	*Yam mild mosaic virus*	YMMV	JX470965	AFV70811.1	1	0	0	0	0	0	0	1	1
Potyviridae	*Potyvirus*	*Yam mosaic virus*	YMV	U42596	YP_022751.1	1	0	0	0	0	0	0	1	1
Potyviridae	*Potyvirus*	*Yambean mosaic virus*	YBMV	JN190431	AEV45179.1	1	0	0	0	0	0	0	1	1
Potyviridae	*Potyvirus*	*Zantedeschia mild mosaic virus*	ZaMMV	AY626825	AAV54595.4	1	0	0	0	0	0	0	1	1
Potyviridae	*Potyvirus*	*Zea mosaic virus*	IJGMV	JQ692088	AFU52934.1	1	0	0	0	0	0	0	1	1
Potyviridae	*Potyvirus*	*Zucchini shoestring virus*	ZSV	KU355553	ANH22633.1	1	0	0	1	0	0	0	1	1
Potyviridae	*Potyvirus*	*Zucchini tigre mosaic virus*	ZTMV	KC345607	AGY36217.1	1	0	0	1	0	0	0	1	1
Potyviridae	*Potyvirus*	*Zucchini yellow mosaic virus*	ZYMV	AF127929	AAD44684.2	1	0	0	0	0	0	0	1	1
Potyviridae	*Roymovirus*	*Passiflora edulis symptomless virus*	PeSV	MH379332	AXL95770.1	0	1	0	0	0	0	0	1	1
Potyviridae	*Roymovirus*	*Rose yellow mosaic virus*	RYMV	JF280796	AFV61646.1	0	1	0	0	0	0	0	1	1
Potyviridae	*Rymovirus*	*Agropyron mosaic virus*	AgMV	AY623626	AAS77619.2	1	0	0	0	0	0	0	1	1
Potyviridae	*Rymovirus*	*Hordeum mosaic virus*	HoMV	AY623627	AAS65455.2	1	0	0	0	0	0	0	1	1
Potyviridae	*Rymovirus*	*Ryegrass mosaic virus*	RGMV	Y09854	CAA70983.1	1	0	0	0	0	0	0	1	1
Potyviridae	*Tritimovirus*	*Brome streak mosaic virus*	BrSMV	Z48506	CAA88417.1	0	1	0	0	0	0	0	1	1
Potyviridae	*Tritimovirus*	*Oat necrotic mottle virus*	ONMV	AY377938	AAQ91884.1	0	1	0	0	0	0	0	1	1
Potyviridae	*Tritimovirus*	*Tall oatgrass mosaic virus*	TOgMV	KF260962	AGZ02786.1	0	1	0	0	0	0	0	1	1
Potyviridae	*Tritimovirus*	*Wheat eqlid mosaic virus*	WEqMV	EF608612	ABU98333.1	0	1	0	0	0	0	0	1	1
Potyviridae	*Tritimovirus*	*Wheat streak mosaic virus*	WSMV	AF057533	AAC13692.1	0	1	0	0	0	0	0	1	1
Potyviridae	*Tritimovirus*	*Yellow oat grass mosaic virus*	YOgMV	KF984546	AIE45539.1	0	1	0	0	0	0	0	1	1
Potyviridae	*Unassigned*	*Common reed chlorotic stripe virus*	CRCSV	KY612317	AQT26513.1	1	0	0	0	0	0	0	1	1
Potyviridae	*Unassigned*	*Longan witches broom-associated virus*	LWBD	KY649478	ARS65734.1	0	1	0	0	0	0	0	1	1
Potyviridae	*Unassigned*	*Spartina mottle virus*	SpMoV	MN788417	QIQ28451.1	1	0	0	0	0	0	0	1	1
TOTAL	–	–	–	–	–	150	20	4	7	2	3	5	181	185

[a] Enzyme signatures of NIa-pro, NIb, and CI were used to map the core proteome

Table S3. Taxonomy and accession numbers of the viruses depicted in Figure 11

Phylum	Order	Family	Genus	Species	Virus_name	Virus_nam	Genome_accession(s)	Host_source
Kitrinoviricota	*Martellivirales*	*Closteroviridae*	*Closterovirus*	*Beet yellows virus*	beet yellows virus	BYV	NC_001598.1	plants
Kitrinoviricota	*Martellivirales*	*Closteroviridae*	*Closterovirus*	*Citrus tristeza virus*	citrus tristeza virus	CTV	U16304.1	plants
Kitrinoviricota	*Martellivirales*	*Closteroviridae*		*Actinidia virus 1*	actinidia virus 1	AcV1	NC_035453.1	plants
Kitrinoviricota	*Amarillovirales*	*Flaviviridae*	*Pestivirus*	*Pestivirus A*	bovine viral diarrhea virus 1	BVDV1	M96751.1	vertebrates
Kitrinoviricota	*Tolivirales*	*Luteoviridae*	*Enamovirus*	*Pea enation mosaic virus 1*	pea enation mosaic virus 1	PEMV1	NC_003629.1	plants
Kitrinoviricota	*Tolivirales*	*Luteoviridae*	*Polerovirus*	*Potato leafroll virus*	potato leafroll virus	PLRV	NC_001747.1	plants
Pisuviricota	*Durnavirales*	*Hypoviridae*	*Hypovirus*	*Cryphonectria hypovirus 1*	*Cryphonectria* hypovirus 1	CHV1	M57938.1	fungi
Pisuviricota	*Durnavirales*	*Hypoviridae*	*Hypovirus*	*Cryphonectria hypovirus 4*	*Cryphonectria* hypovirus 4	CHV4	MK533145.1	fungi
Pisuviricota	*Nidovirales*	*Arteriviridae*	*Alphaarterivirus*	*Alphaarterivirus equid*	equine arteritis virus	EAV	NC_002532.2	vertebrates
Pisuviricota	*Nidovirales*	*Arteriviridae*	*Deltaarterivirus*	*Deltaarterivirus hemfev*	simian hemorrhagic fever virus	SHFV	NC_003092.2	vertebrates
Pisuviricota	*Nidovirales*	*Arteriviridae*	*Betaarterivirus*	*Betaarterivirus suid 2*	porcine reproductive and respiratory syndrome virus 2	PRRSV-2	AY150564.1	vertebrates
Pisuviricota	*Nidovirales*	*Coronaviridae*	*Alphacoronavirus*	*Porcine epidemic diarrhea virus*	porcine epidemic diarrhea virus	PEDV	NC_003436.1	vertebrates
Pisuviricota	*Nidovirales*	*Coronaviridae*	*Betacoronavirus*	*Severe acute respiratory syndrome-related coronavirus*	severe acute respiratory syndrome coronavirus 2	SARS-Co\	NC_045512.2	vertebrates
Pisuviricota	*Picornavirales*	*Picornaviridae*	*Anativirus*	*Anativirus A*	anativirus A1; duck picornavirus	AnaV-A1	NC_006553.1	vertebrates
Pisuviricota	*Picornavirales*	*Picornaviridae*	*Aphthovirus*	*Foot-and-mouth disease virus*	foot-and-mouth disease virus O	FMDV	NC_039210.1	vertebrates
Pisuviricota	*Picornavirales*	*Picornaviridae*	*Cardiovirus*	*Cardiovirus A*	cardiovirus A1; encephalomyocarditis virus 1	EMCV-1	M81861.1	vertebrates
Pisuviricota	*Picornavirales*	*Picornaviridae*	*Cardiovirus*	*Cardiovirus B*	cardiovirus B1; Theiler's murine encephalomyeltits virus	TMEV	NC_001366.1	vertebrates
Pisuviricota	*Picornavirales*	*Picornaviridae*	*Enterovirus*	*Enterovirus B*	echovirus 7	EV7	AY302559.1	vertebrates
Pisuviricota	*Picornavirales*	*Secoviridae*	*Fabavirus*	*Broad bean wilt virus 1*	broad bean wilt virus 1	BBWV1	NC_005289.1, NC_005290.1	plants
Pisuviricota	*Picornavirales*	*Secoviridae*	*Waikavirus*	*Maize chlorotic dwarf virus*	maize chlorotic dwarf virus	MCDV	NC_003626.1	plants
Pisuviricota	*Sobelivirales*	*Solemoviridae*	*Sobemovirus*	*Cocksfoot mottle virus*	cocksfoot mottle virus	CfMV	NC_002618.2	plants
Pisuviricota	*Patatavirales*	*Potyviridae*	*Arepavirus*	*Areca palm necrotic spindle-spot virus*	areca palm necrotic spindle-spot virus	ANSSV	NC_040836.1	plants
Pisuviricota	*Patatavirales*	*Potyviridae*	*Brambyvirus*	*Blackberry virus Y*	blackberry virus Y	BlVY	NC_008558.1	plants
Pisuviricota	*Patatavirales*	*Potyviridae*	*Ipomovirus*	*Cucumber vein yellowing virus*	cucumber vein yellowing virus	CVYV	NC_006941.1	plants
Pisuviricota	*Patatavirales*	*Potyviridae*	*Ipomovirus*	*Ugandan cassava brown streak virus*	Ugandan cassava brown streak virus	UCBSV	FJ185044.1	plants
Pisuviricota	*Patatavirales*	*Potyviridae*	*Macluravirus*	*Chinese yam necrotic mosaic virus*	Chinese yam necrotic mosaic virus	CYNMV	NC_018455.1	plants
Pisuviricota	*Patatavirales*	*Potyviridae*	*Poacevirus*	*Triticum mosaic virus*	*Triticum* mosaic virus	TriMV	NC_012799.1	plants
Pisuviricota	*Patatavirales*	*Potyviridae*	*Potyvirus*	*Plum pox virus*	plum pox virus	PPV	AJ243957.1	plants
Pisuviricota	*Patatavirales*	*Potyviridae*	*Potyvirus*	*Sweet potato feathery mottle virus*	sweet potato feathery mottle virus	SPFMV	NC_001841.1	plants
Pisuviricota	*Patatavirales*	*Potyviridae*	*Tritimovirus*	*Wheat streak mosaic virus*	wheat streak mosaic virus	WSMV	NC_001886.1	plants

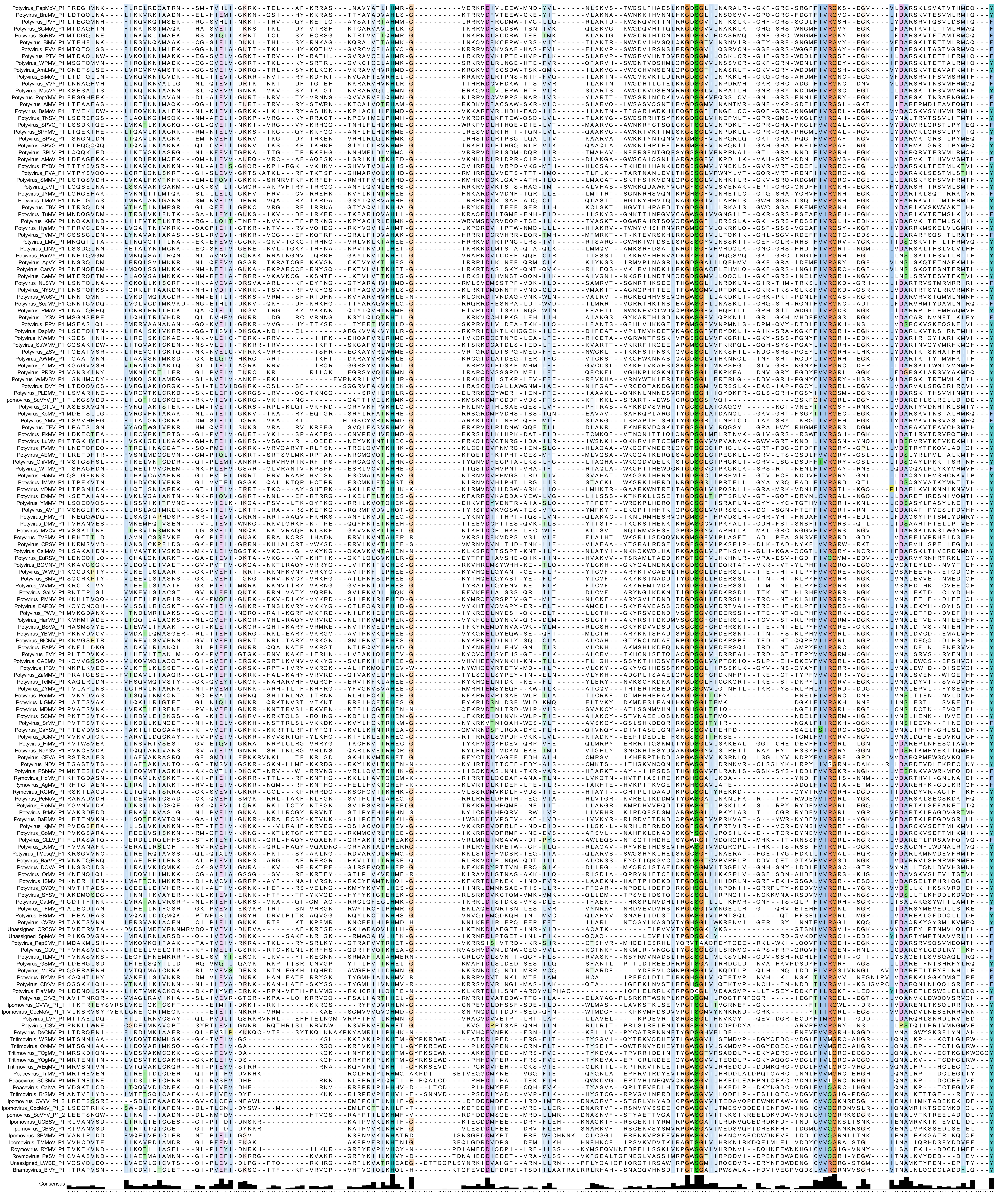

Figure S1. Alignment of P1 protease domains.

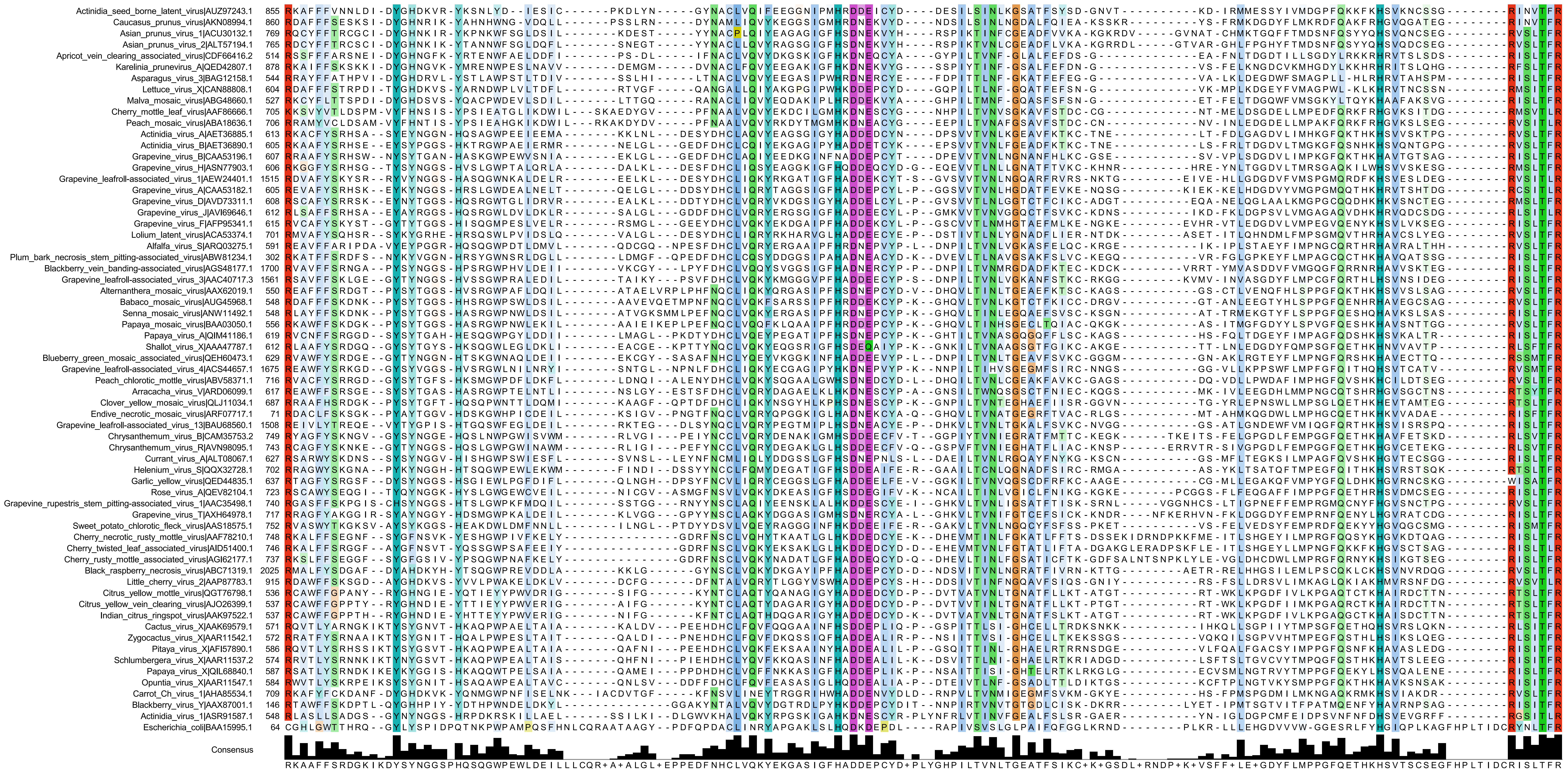

Figure S2. Alignment of AlkB domains.

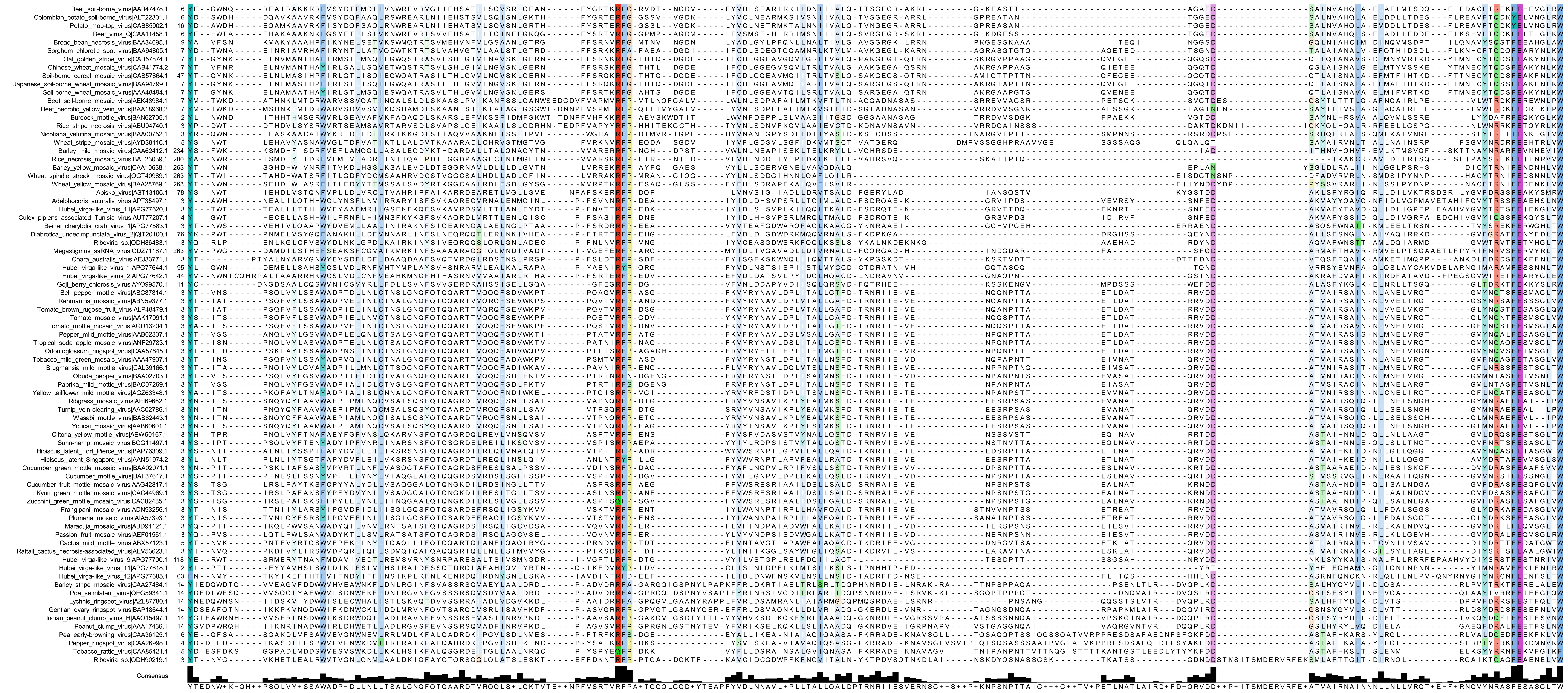

Figure S3. Alignment of TMV-like CP domains.

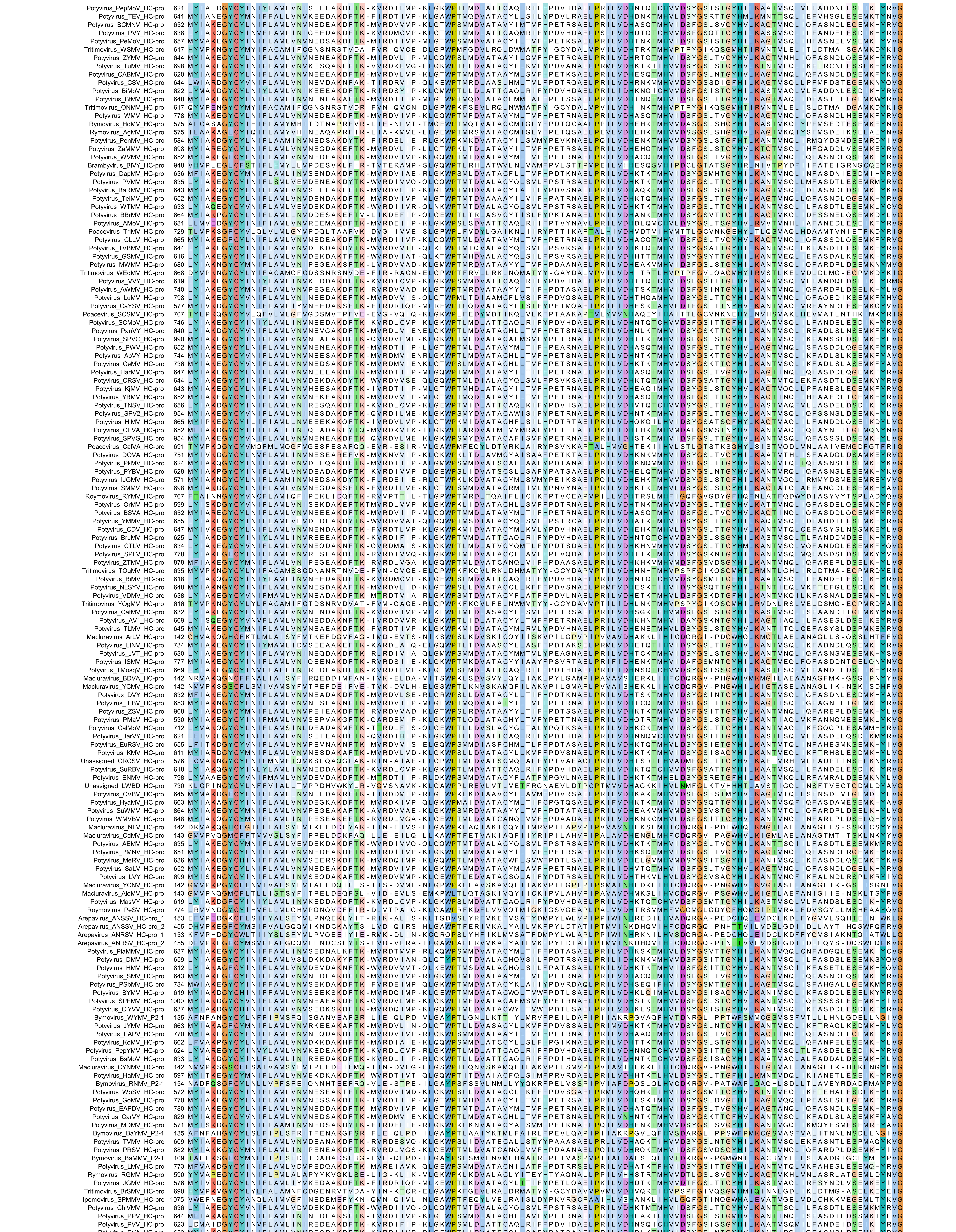

Figure S4. Alignment of HC-pro protease domains.

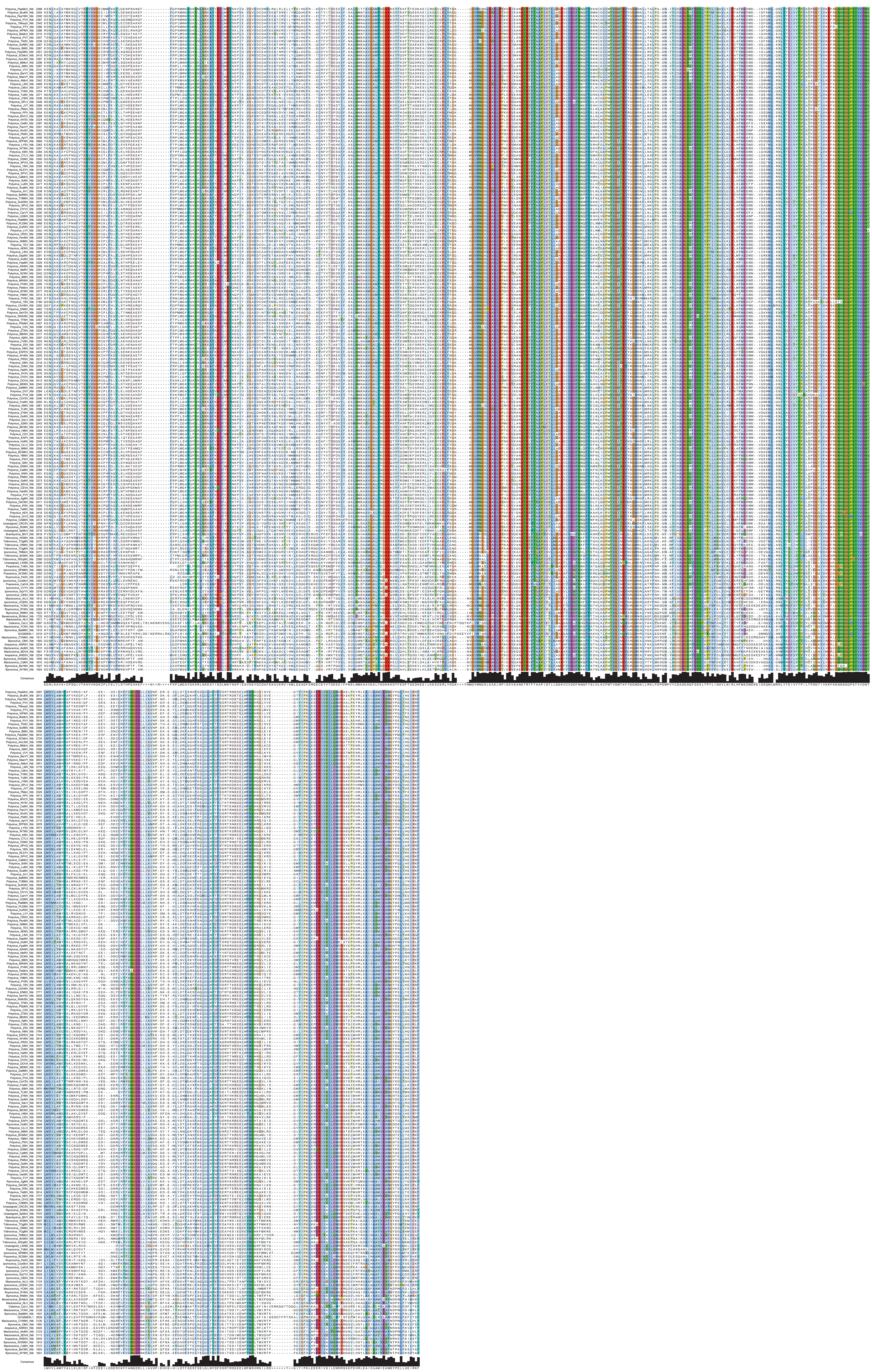

Figure S5. Alignment of RdRp domains.
